# Structural Expansion of Dibenzo[*b*,*d*]thiophene Sulfone through Functionalization at Bay Positions

**DOI:** 10.1021/acs.orglett.5c02414

**Published:** 2025-08-12

**Authors:** Zuzanna Spychalska, Jan Adamek, Włodzimierz Buchowicz, Krzysztof Durka, Sergiusz Luliński

**Affiliations:** Warsaw University of Technology, Faculty of Chemistry, Noakowskiego 3, 00-664 Warsaw, Poland

## Abstract

We report a synthesis of 1,9-dibromodibenzo­[*b*,*d*]­thiophene sulfone, which was converted to a respective dilithio intermediate. Subsequent trapping with electrophiles afforded either 1-substituted (R = CHO, B­(OH)_2_) or 1,9-disubstituted (R = CO_2_H, I) derivatives, indicating an effect of steric factors on the outcome of this reaction. Various annulation reactions were also probed giving rise to extended π-conjugated systems. The electronic and optical properties of all compounds were characterized by cyclic voltammetry, UV–vis spectroscopy, and DFT calculations.

## Graphical abstract

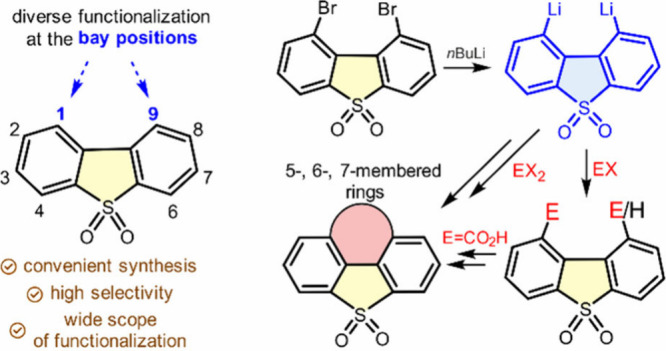

Dibenzo­[*b*,*d*]­thiophene sulfone (DBTS) is a rigid and thermally stable building block with inherent electron-acceptor properties. Both discrete molecular systems as well as various conjugated oligomers or polymers featuring the DBTS moiety have found numerous applications in materials chemistry, mostly due to their promising photophysical properties. Reported examples include the use of DBTS derivatives as luminescent molecules (in particular, thermally activated delayed fluorescent emitters, TADF),^1^ photocatalysts for H_2_ generation,^2^ and solar cell materials.^3^ DBTS derivatives were also used for the synthesis of microporous polymers^4^ and studied in the context of crystal engineering.^5^ The synthesis of those materials often involves the use of symmetrical dibromo DBTS derivatives. The well-known 3,7-isomer (Scheme 1) can be obtained by aromatic electrophilic substitution,^6^ whereas the 2,8-derivative is accessible through dibromination of dibenzo­[*b*,*d*]­thiophene followed by dioxygenation of the sulfur atom.^7^ In turn, the 4,6-dibromo derivative has been synthesized only recently and used as an acceptor unit of blue and green TADF D–A–D emitters.^8^

**Scheme 1:**
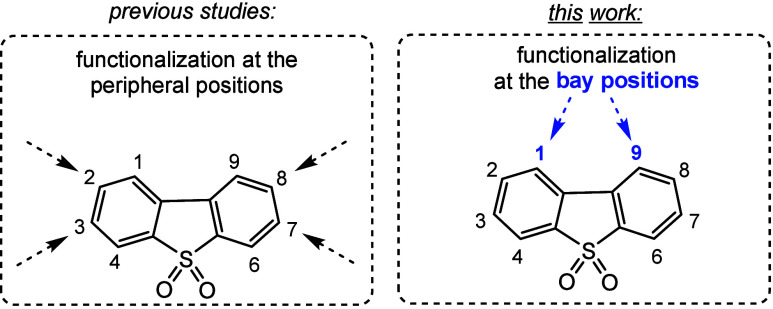
Structure of Dibenzo­[*b*,*d*]­thiophene Sulfone with the Atom Numbering Scheme

Herein, we report the synthesis of 1,9-dibromodibenzo­[*b*,*d*]­thiophene sulfone **2,** which fills the gap concerning DBTS functionalization at the bay (that is, 1,9) positions. In the first step, bis­(3-bromophenyl)­sulfone **1** was subjected to a double regioselective deprotonation with LDA (2.2 equiv) in THF at ca. −85 °C at the positions between the bromine and sulfonyl substituents (Scheme 2). Subsequently, the addition of CuCl_2_ resulted in the formation of **2** in 77% yield.

**Scheme 2:**
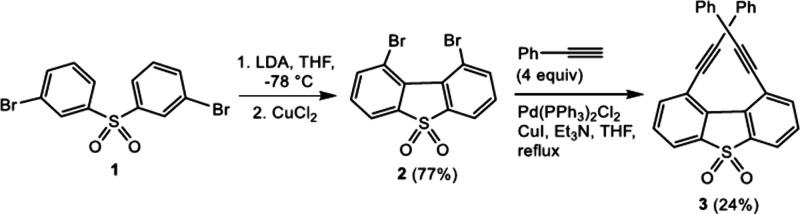
Synthesis of 1,9-Dibromodibenzo­[*b*,*d*]­thiophene Sulfone **2** and the Subsequent Sonogashira Coupling Affording Bis­(Phenylethynyl) Derivative **3**

Single crystal X-ray diffraction analysis revealed that the molecular structure of **2** is characterized by a distortion of the DBTS core from planarity (Figure 1a) as the dihedral angle τ defined by four carbon atoms in the bay region is 31.8°. The observed angular strain is caused by repulsive interactions of the Br atoms as the intramolecular Br···Br contact of 3.354 Å is shorter than the sum of the respective van der Waals radii (3.66 Å). Compound **2** crystallizes in the *P*2_1_/*c* space group, indicating that each single crystal comprises a racemic mixture of two helical enantiomers. However, according to DFT (B3LYP/6–311++G­(d,p)) calculations, the inversion barrier between them is only 26 kJ·mol^–1^, indicating that **2** is configurationally labile in solution at room temperature. Moreover, calculations revealed that **2** is 64 kJ·mol^–1^ less stable compared to the 3,7-dibromo isomer (also bearing Br atoms at the meta positions with respect to the SO_2_ group), reflecting the internal strain in the former system. In the consecutive step, compound **2** was employed for the synthesis of several DBTS derivatives, mostly those featuring an extended π-conjugation. Initially, a double Sonogashira coupling with phenylacetylene^5^ gave rise to 1,9-bis­(phenylethynyl)­dibenzo­[*b*,*d*]­thiophene sulfone **3**, but the isolated yield was rather low (24%).

**Figure 1:**
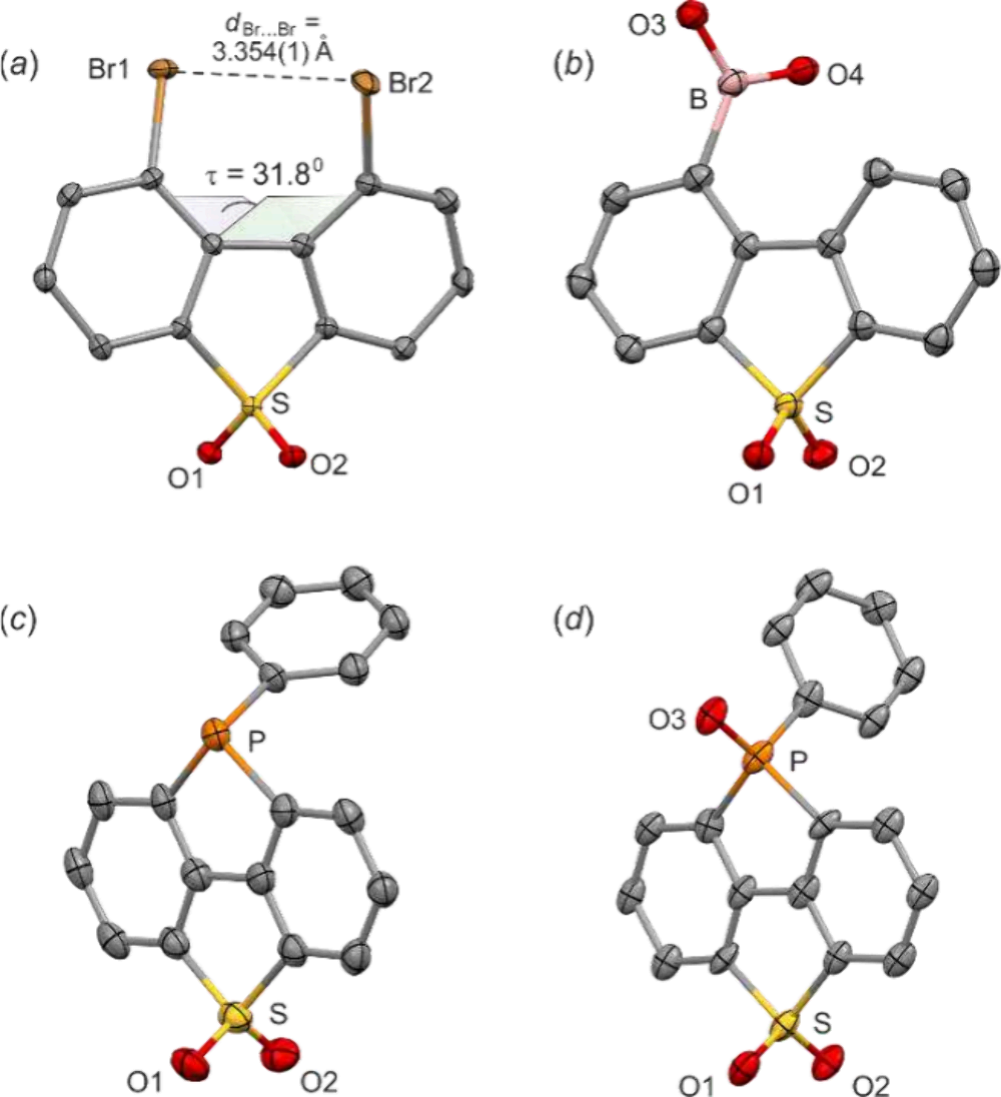
Molecular structures of (*a*) **2**, (*b*) **5**, (*c*) **12**, (*d*) **13**. Thermal motions are given as ADPs at the 50% probability level. Hydrogen atoms are omitted for clarity.

Next, we turned our attention to the approach based on the generation of 1,9-dilithio intermediate **2-Li**_**2**_ followed by treatment with appropriate electrophiles (Scheme 3). The reaction of **2** with *n*-BuLi (2.2 equiv) in THF/Et_2_O at −90 °C resulted in a double Br/Li exchange, giving rise to an olive-brown solution of **2-Li**_**2**_. The structure of the monomeric solvate **2-Li**_**2**_·(THF)_4_, assumed to be one of the possible structures, was modeled by DFT calculations. It was found that the Li atoms are located on the symmetry plane bisecting the perfectly planar DBTS backbone with the Li···Li distance of 2.59 Å (Figure S6, Supporting Information). Thus, it is plausible that the structural relaxation may be an additional driving force for the effective lithiation. Importantly, we did not observe isomerization to the 4,6-dilithio derivative, which could potentially occur due to the higher acidity of 4,6-H atoms. The intermediate **2-Li**_**2**_ was successfully used for the synthesis of various DBTS derivatives upon reactions with the appropriate electrophiles. Interestingly, monosubstituted derivatives, i.e., the aldehyde **4** and boronic acid **5**, were isolated exclusively using DMF and B­(O*i*Pr)_3_, whereas the reactions with I_2_ and CO_2_ gave rise to 1,9-disubstituted diiodo- and di­(carboxylic acid) derivatives **6** and **7**, respectively. This can be ascribed to the proximity of 1 and 9 positions, which effectively hampers the reaction of the second C–Li bond with bulkier electrophiles. It should be noted that the molecular structure of **5** was determined by single crystal X-ray diffraction (Figure 1b). This compound also shows an interesting supramolecular structure resulting from hydrogen-bonding interactions of the B­(OH)_2_ and SO_2_ groups (for details, see the Supporting Information).

**Scheme 3:**
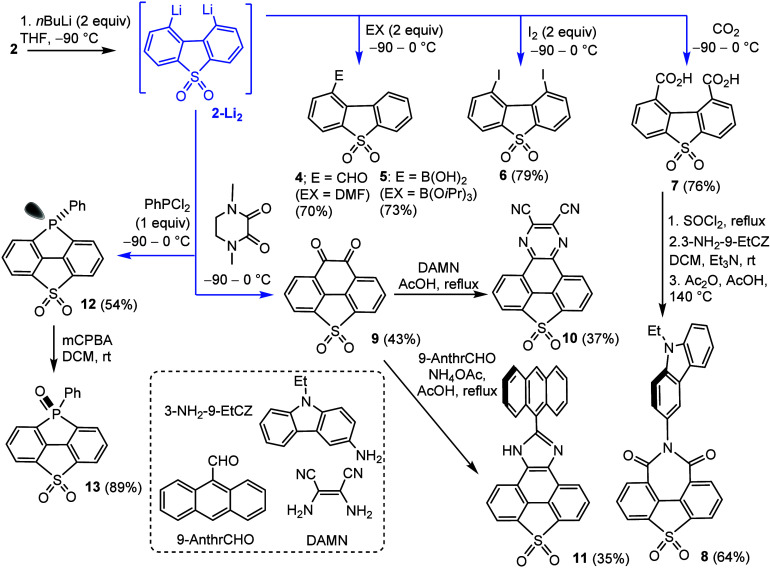
Generation of 1,9-Dilithiodibenzo­[*b*,*d*]­thiophene Sulfone **2-Li_2_** and Subsequent Conversion to Products **4–13**

We also subjected **2-Li**_**2**_ to the annulation reaction with *N*,*N*′-dimethylpiperazine-2,3-dione^9^ resulting in the formation of compound **9** in 43% yield (Scheme 3). It can be regarded as the SO_2_-bridged analogue of the phenanthrene *ortho*-quinone. We also probed the synthesis of some derivatives featuring extended π-conjugation. Thus, compound **7** was converted into imide **8** bearing *N*-ethylcarbazol-3-yl as a donor moiety attached to the bridging N atom. Compound **9** was subjected to the cyclocondensation with diaminomaleonitrile (DAMN)^10^ producing compound **10** comprising another strongly electron-acceptor 2,3-dicyanopyrazine-5,6-diyl moiety attached at the 1,9-positions of the DBTS core. In a different approach, the cyclocondensation of **9** with 9-anthraldehyde in the presence of NH_4_OAc in AcOH^11^ afforded the imidazole-fused derivative **11**. Finally, treatment of **2-Li**_**2**_ with PhPCl_2_ afforded strained cyclic phosphine **12** in 54% yield. This compound was readily converted to corresponding P-oxide **13**. Compounds **12** and **13** belong to the class of bis­(heteroatom)-bridged biphenyls, characterized by their compact and planar structure. The structural specificity of **12** and **13** was confirmed by single-crystal X-ray diffraction data (Figure 1c, d). It is manifested by the elongation of the endocyclic P–C bond distances (**12**: *d*_P–C_ = 1.853(6) and 1.864(6) Å; **13**: *d*_P–C_ = 1.844(5) and 1.855(5) Å) with respect to the exocyclic ones (**12**: 1.821(6) Å; **13**: 1.797(6) Å). The sum of the C–P–C angles in **13** is higher (307.5°) compared to **12** (297.5°), reflecting the increased sp^3^ hybridization at the P atom. In addition, the central C–C bonds are much shorter (**12**: 1.426(8) Å; **13**: 1.439(7) Å) in comparison to that in dibenzophosphole oxide (*d*_C1–C1′_ = 1.480 Å).^12^ Overall, the geometric parameters of **13** are comparable with those found in related bis­(PhPO)-bridged biphenyls.^13^ Notably, the ^31^P NMR resonances of **12** (18.6 ppm) and **13** (43.8 ppm) are strongly deshielded with respect to those observed for analogues comprising the 5-membered phosphole ring, namely 5-phenyldibenzophosphole and its oxide (δ^31^P = −11.1 and 29.1 ppm,^14^ respectively). The difference of ^31^P NMR chemical shifts is even larger when comparing **12** and related 10-phenyl-10*H*-9-thia-10-phosphaanthracene-9,9-dioxide (δ^31^P = −17.3 ppm).^15^ On the other hand, the ^31^P NMR characteristics of **12** and **13** are in line with those of bis­(PhP)- and bis­(PhPO)-bridged biphenyls,^13^ which strongly suggests that the structure strain is responsible for the observed ^31^P nucleus deshielding effects.

Apart from the characterization of all compounds **2–13** using NMR spectroscopy and HRMS, their molecular geometries were optimized using DFT calculations (Figures S3–S5). The geometric parameters for **2**, **5**, **12**, and **13** are in good agreement with those derived from crystallographic studies. The DBTS core is significantly distorted in the optimized geometries of **2** (*vide supra*), **3**, **6**, and **7** (Table S3). However, as mentioned above for **2**, the interconversion barrier between the respective helical enantiomers is far too low (at best, 46 kJ·mol^–1^ for **6**) for their resolution (Table S4). Accordingly, attempts to resolve **7** on a CHIRALPAK IC column were not successful.

The characterization of the electronic properties of **2–13** was performed using cyclic voltammetry measurements. The results are collected in Table S6. For the majority of compounds, only reduction potentials could be measured due to the lack of oxidizable units in their structures. The corresponding electron affinity (EA) values span a wide range. Not surprisingly, the 1,2-dione **9** derivative is the strongest acceptor with the high EA of 4.19 eV. Compounds **2**–**3**, **6**–**8**, and **10**, containing other electron-withdrawing moieties, are also effective acceptors with the EA values in the range of 3.21–3.69 eV. The angular strain contributes to the acceptor potency as shown by the comparison of EA for **2** vs **6** (3.21 and 3.29 eV, respectively). Even though iodine is less electronegative than bromine, the EA of **6** is higher, which is in line with the stronger deformation of the DBTS core in **6** vs **2** as indicated by a DFT-computed structure with the larger bay torsion angle τ = 35.0° and higher destabilization (by 75 kJ·mol^–1^ relative to the 3,7-diiodo-isomer). Apart from the extended π-conjugation with phenylethynyl substituents, a similar effect may contribute to a high EA for **3** (3.51 V) where the bay torsion angle is 24.7°. The electrochemical studies were complemented by DFT calculations of frontier orbital energies (Table S5) and their spatial distribution (Figures S7–S9). In most cases, LUMOs are delocalized throughout the DBTS core, while their energies roughly correspond to the EA values. Finally, optical properties of selected derivatives were investigated using UV–vis absorption and emission spectroscopy (Table 1, Figure S12). Normalized emission spectra of luminescent systems are shown in Figure 2. Compounds **3**, **10**, and **11** display blue and green emission with moderate to low quantum yield values (Φ_PL_). All compounds exhibit monoexponential fluorescence decay, with typical fluorescence lifetimes in the nanosecond range. According to the theoretical calculation, HOMO and LUMO orbitals are delocalized over the entire molecule **3**. For **10**, the observed emission possesses charge transfer character resulting from the electron transition from dibenzo­[*b*,*d*]­thiophene sulfone to the 2,3-dicyanopyrazine-5,6-diyl moiety. For compound **11**, it predominantly occurs within the anthracene subunit.

**Figure 2:**
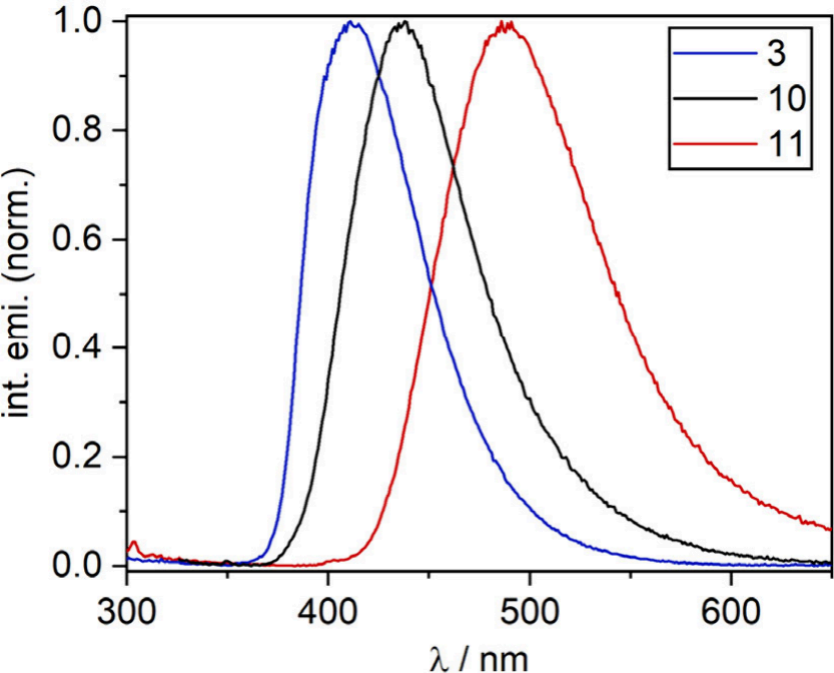
Normalized emission spectra of compounds **3**, **10**, and **11**.

**Table 1:** Spectral Properties of Compounds **3**, **10**, and **11**

	λ_abs_/nm (Σ/10^4^ M^–1^ cm^–1^)	λ_emi_/nm	Φ_PL_/%	τ/ns
**3** ^a^	282 (9.8)	411	18.2	1.9
**10** ^a^	250 (4.6), 278 (5.2), 315 (3.0)	438	3.7	1.5
**11** ^b^	260 (17.7), 281 (9.7), 331 (3.5), 399 (1.8)	491	4.0	3.7

a Spectra measured in CHCl_3_ (10^–5^ M).

b Spectrum measured in DMSO (10^–5^ M).

In conclusion, we have developed a simple and effective protocol for the functionalization of the DBTS scaffold based on the high yield synthesis of 1,9-dibromo derivative **2** followed by the effective generation of the 1,9-dilithio derivative as the key intermediate. Subsequent transformations afforded various derivatives, including some annulated systems featuring different electronic properties due to the presence of the additional electron-donating or -withdrawing moieties. The analysis of collected electrochemical and computational data allowed us to draw conclusions regarding the relationship between the structure and electron-acceptor properties of obtained compounds. The effect of angular strain, manifested by deformation of the DBTS backbone, was also highlighted in this context. Furthermore, compounds **3**, **10**, and **11** are photoluminescent, which confirms the potential of presented methodology for the synthesis of new optical materials. Further studies aimed mainly at the application and extension of the presented general protocol toward the synthesis of new effective emitters for the potential use in TADF-OLEDs are currently underway, and the results will be given in due course.

## Supplementary Material





## Data Availability

The data underlying this study are available in the published article and its .

## Notes

Experimental procedures, spectroscopic data for new compounds, copies of NMR spectra, theoretical calculation details, UV–vis spectra, cyclic voltammetry, and X-ray crystallographic data (PDF)
Theoretical structures (XYZ)

## Notes

E-mail: zuzanna.spychalska.stud@pw.edu.pl (Z.S.); E-mail: jan.adamek.dokt@pw.edu.pl (J.A.); E-mail: wlodzimierz.buchowicz@pw.edu.pl (W.B.); E-mail: krzysztof.durka@pw.edu.pl (K.D.)
