# Supplementary material for: Structural Expansion of Dibenzo[b,d]thiophene Sulfone through Functionalization at Bay Positions
Source: Org Lett. 2025 Aug 12;27(33):9136–40. doi: 10.1021/acs.orglett.5c02414 (PMC12379161; doi:10.1021/acs.orglett.5c02414)
Supplement: Supplementary file 1 [file ol5c02414_si_001.pdf]

Structural expansion of dibenzo[*b*,*d*]thiophene sulfone through functionalization at bay positions

Zuzanna Spychalska, Jan Adamek, Włodzimierz Buchowicz, Krzysztof Durka, Sergiusz Luliński*

Warsaw University of Technology, Faculty of Chemistry, Noakowskiego 3, 00-664 Warsaw, Poland

Supporting Information

List of contents

Synthesis ………………………………………………………………………….…… S2

Single crystal X-ray crystallography …………………………………………….......... S10

Theoretical calculations ……………………………………………………….......... S13

Electrochemistry ……………………………………………………………….....… S22

UV-Vis spectroscopy ………………………………………………………………… S26

References …………………………………………………………………………… S27

NMR spectra ...…………………………………………………………………….. S29

HRMS spectra ……………………………………………………………….... S41

Synthesis

General comments. Solvents used for reactions were dried by heating to reflux (oil bath) with sodium/benzophenone and distilled under argon. Starting materials and other reagents were used as received without further purification. Reactions involving organometallic compounds were carried out under an argon atmosphere. ^{1}H, ^{13}C, and ^{31}P NMR spectra were recorded on an Agilent NMR 400 MHz DDR2 or JEOL 600 MHz spectrometers. ^{1}H and ^{13}C NMR chemical shifts are given relative to TMS using residual solvent resonances. ^{31}P NMR chemical shifts are given relative to 85% phosphoric acid solution in D_2O. High-resolution Mass Spectra were recorded using a Synapt G2-S HDMS mass spectrometer (Waters) equipped with an atmospheric-pressure chemical ionization (APCI) and electrospray (ESI) ion sources, and a quadrupole-time-of-flight (q-TOF) mass analyzer.

Bis(3-bromophenyl)sulfone (**1**).

Method A:

Compound **1** was obtained according to the published procedure[1] with modification of the workup step: a mixture of diphenyl sulfone (10.9 g, 0.05 mol), *N*-bromosuccinimide (18.7 g, 0.105 mol), and H_2SO_4 (50 mL) was heated at 100 °C for 2 h (**Scheme S1**). The mixture was poured onto ice (200 g). An oil was separated and washed several times with water. It was diluted in $CHCl_3$ (10 mL) followed by the addition of Et_2O (80 mL) resulting in the precipitation of the product which was filtered, washed with Et_2O (3 × 10 mL) and dried. A white solid was obtained. Yield 4.8 g (26%). ^{1}H NMR (400 MHz, $CDCl_3$) δ 8.08 (t, J = 1.8 Hz, 2H), 7.88–7.86 (m, 2H), 7.73–7.71 (m, 2H), 7.41 (t, J = 7.9 Hz, 2H) ppm. ^{1}H NMR spectrum was in agreement with the reported data.[1]

Method B:

To a solution of 1,3-dibromobenzene (29.4 g, 125 mmol) in THF (80 mL), *i*-PrMgCl·(2 M in THF, 65 ml, 130 mmol) was added dropwise at rt during 15 min. The temperature increased to ca. 35 °C and the mixture was stirred at rt for 4 h. It was cooled to −70 °C and a solution of $SOCl_2$ (4.7 mL, 65 mmol) in Et_2O (20 mL) was added dropwise during 40 min, maintaining temperature below −20 °C. After 30 min a cooling bath was removed and a mixture was allowed to warm up to an ambient temperature and stirred overnight. Et_2O (100 mL) was added and a mixture was quenched with water (50 mL). The organic layer was separated and the aqueous layer was washed with Et_2O (50 mL). Combined organic layers were washed

with brine (100 mL) and dried over anhydrous $MgSO_4$. The solution was filtered and concentrated in vacuo to give crude bis(3-bromophenyl)sulfoxide as a yellow oil. ^{1}H NMR (600 MHz, $CDCl_3$) δ 7.80 (t, J = 1.8 Hz, 1H), 7.59 (ddd, J = 7.9, 1.9, 1.0 Hz, 1H), 7.56 (ddd, J = 7.8, 1.7, 1.0 Hz, 1H), 7.35 (t, J = 7.9 Hz, 1H) ppm. $^{13}C\{^{1}H\}$ NMR (151 MHz, $CDCl_3$) δ 147.5, 134.7, 131.1, 127.5, 123.8, 123.3 ppm. It was dissolved in DCM (50 mL) and mCPBA (77%, 18.0 g, 80 mmol) was added in a few portions at 0 °C. After a few minutes the cooling bath was removed and a mixture was allowed to warm up to the room temperature. A mixture was stirred overnight at the room temperature; a white precipitate was formed. An aq. $Na_2S_2O_5$ solution (10wt%, 50 mL) was added and the mixture was stirred for 30 min. A mixture was diluted with DCM (50 mL) and aq. 1 M NaOH solution was added to reach pH ≈ 10 and to dissolve a remaining solid. The organic phase was separated, washed with water (50 mL), and dried with anhydrous Na_2SO_4. The mixture was filtered through Celite pad and concentrated under reduced pressure. Et_2O (50 mL) was added to the obtained residue and the suspension was stirred for 1 h and filtered. The solid was washed with Et_2O (2 × 20 mL) and dried to give **1** as a white powder (10.5 g, 45%).

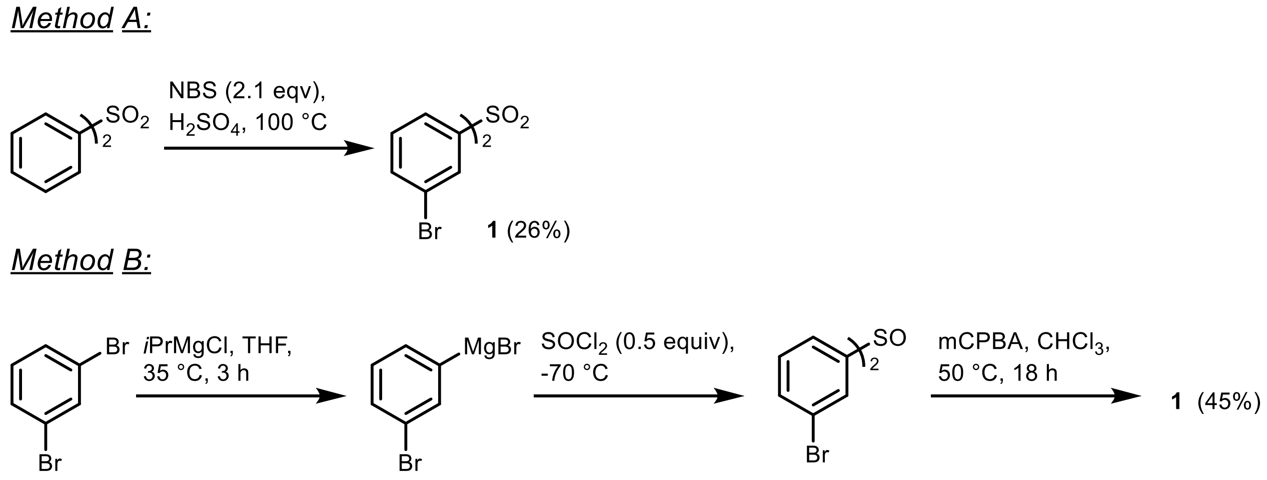

Scheme S1. Synthesis of bis(3-bromophenyl)sulfone (**1**).

1,9-Dibromodibenzo[*b*,*d*]thiophene sulfone (2).

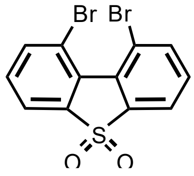

To a solution of LDA (62.5 mmol), prepared from *n*-BuLi (2.5 M in hexane, 25 mL, 62.5 mmol) and diisopropylamine (6.3 g, 63.0 mmol) in THF (50 mL) at −70 °C, a solution of bis(3-bromophenyl)sulfone (11.22 g, 30.0 mmoL) in THF (40 mL) was added dropwise whilst the temperature was maintained at −90 °C. The mixture was stirred at ca. −90 - −85 °C for ca. 30 min resulting in the formation of a beige suspension. Anhydrous $CuCl_2$ (dried for 2 h at 140 °C under vacuum, 8.9 g, 65.0 mmol) was added and the mixture turned dark immediately whilst the temperature increased to ca. -60 °C. The mixture was allowed to warm to the room temperature and hydrolyzed with aq. 2 M HCl (50 mL). Et_2O (100 mL) was added and the organic phase was separated an dried with Na_2SO_4. Solvents were removed under reduced pressure to leave the crude product which was dissolved in a minimum amount of DCM followed by the addition of Celite (ca. 30 g). The solvent was removed under reduced pressure and the remaining powder was placed onto the pad of silica. The product was eluted with DCM (5 × 50 mL). The collected filtrate was concentrated in vacuo and the isolated solid was washed with Et_2O (2 × 20 mL) to give the product as a beige powder. Yield 8.60 g (77%). ^{1}H NMR (400 MHz, $CDCl_3$) δ 7.89 (d, *J* = 8.0 Hz, 2H), 7.84 (d, *J* = 7.5 Hz, 2H), 7.46 (t, *J* = 7.7 Hz, 2H) ppm. ^{13}C{^{1}H} NMR (101 MHz, $CDCl_3$) δ 141.3, 140.6, 131.9, 131.6, 121.1, 119.4 ppm. HRMS (ESI, positive ion mode) m/z: $[M + H]^+$ Calcd for $C_{12}H_7Br_2O_2S$ 372.8528; found 372.8529.

1,9-Bis(phenylethynyl)dibenzo[*b*,*d*]thiophene sulfone (**3**).

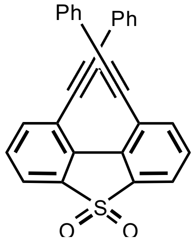

A mixture of compound **2** (374 mg, 1.0 mmol), phenylacetylene (408 mg, 4.0 mmol), CuI (23 mg, 0.12 mmol), $Pd(PPh_3)_2Cl_2$ (42 mg, 0.06 mmol), Et_3N (1.0 mL) and THF (5 mL) was refluxed for 16 h under argon atmosphere. It was cooled to rt and 2 M aq. HCl (5 mL) was added. The mixture was extracted with DCM (2 × 20 mL). The extract was dried with Na_2SO_4 and concentrated under reduced pressure. The residue was subjected to column chromatography on silica (eluent hexane/DCM). The product was isolated as a white solid. Yield 99 mg (24%). ^{1}H NMR (600 MHz, $CDCl_3$) δ 7.89 (dd, *J* = 7.8, 1.2 Hz, 2H), 7.83 (dd, *J* = 7.5, 1.2 Hz, 2H), 7.58 (t,*J* = 7.7 Hz, 2H), 7.37–7.34 (m, 4H), 7.25–7.22 (m, 2H), 7.20–7.16 (m, 4H) ppm. ^{13}C{^{1}H} NMR (600 MHz, $CDCl_3$) (151 MHz, $CDCl_3$) δ 139.6, 139.5, 132.3, 131.7, 130.0, 128.9, 128.3, 122.8, 121.9, 121.4, 101.6, 88.5 ppm. HRMS (ESI, positive ion mode) m/z: $[M + H]^+$ Calcd for $C_{28}H_{17}O_2S$ 417.0944; found 417.0942.

Dibenzo[*b*,*d*]thiophene-1-carbaldehyde sulfone (**4**).

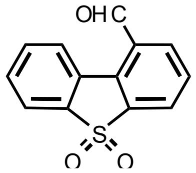

A solution of **2** (0.38 g, 1.0 mmol) in THF (5 mL) was added dropwise to a solution of n-BuLi (2.5 M in hexane, 1.0 mL, 2.5 mmol) in THF (10 mL) and Et_2O (10 mL) at −90 °C. The resulting olive-brown solution was stirred at −90 °C for ca. 30 min followed by the addition of a solution of DMF (0.25 mL, 3.0 mmol) in Et_2O (3 mL). The mixture was allowed to warm to the room temperature and hydrolyzed with aq. 2 M HCl (5 mL). Et_2O (20 mL) was added and the organic phase was separated. Solvents were removed under reduced pressure to leave the crude product which was washed with Et_2O (2 × 5 mL) to give the product as a cream white powder. Yield 181 mg (70%). ^{1}H NMR (600 MHz, acetone-d_6) δ 10.68 (s, 1H), 8.64 (d, J = 8.2 Hz, 1H), 8.30 (dd, J = 7.7, 1.2 Hz, 1H), 8.19 (dd, J = 7.6, 1.3 Hz, 1H), 7.99 (dd, J = 7.7, 1.3 Hz, 1H), 7.91 (t, J = 7.7 Hz, 1H), 7.85 (td, J = 7.8, 1.3 Hz, 1H), 7.78 (td, J = 7.5, 1.0 Hz, 1H) ppm. ^{13}C{^{1}H} NMR (151 MHz, acetone-d_6) δ 191.0, 140.0, 138.4, 137.0, 134.57, 134.43, 131.7, 131.3, 130.9, 130.2, 128.4, 126.4, 122.1 ppm. HRMS (ESI, positive ion mode) m/z: $[M + H]^+$ Calcd for $C_{13}H_9O_3S$ 245.0267; found 245.0267.

(5,5-Dioxidodibenzo[*b*,*d*]thiophen-1-yl)boronic acid (**5**).

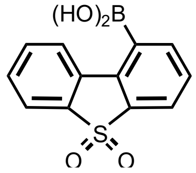

This compound was obtained as described for **4** using B(O*i*Pr)$_3$ (0.75 mL, 3.0 mmol) as the electrophile. The product was obtained as a white powder. Yield 190 g (73%). ^{1}H NMR (400 MHz, acetone-d_6) δ 8.22 (d, J = 7.9 Hz, 1H), 8.05 (s, 2H), 7.90–7.76 (m, 4H), 7.66 (t, J = 7.6 Hz, 1H), 7.62 (t, J = 7.6 Hz, 1H) ppm. ^{13}C{^{1}H} NMR (101 MHz, acetone-d_6) δ 138.7, 138.4, 138.3, 134.6, 133.5, 133.3, 131.0, 130.2, 124.40, 122.38, 122.3 ppm. HRMS (ESI, positive ion mode) m/z: $[M + H]^+$ Calcd for $C_{12}H_{10}BO_4S$ 261.0387; found 261.0388. HRMS (ESI, negative ion mode) m/z: $[M - H]^-$ Calcd for $C_{12}H_8BO_4S$ 259.0242; found 259.0240.

1,9-Diiododibenzo[*b*,*d*]thiophene sulfone (**6**).

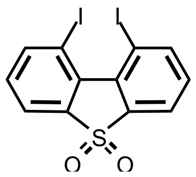

This compound was obtained as described for **4** using iodine (0.76 g, 3.0 mmol) as the electrophile. The hydrolysis was performed using aq. 10 wt% Na_2SO_3 in order to reduce an excess of iodine. Yield 0.37 g (79%). ^{1}H NMR (600 MHz, $CDCl_3$) δ 8.18 (dd, J = 7.9, 1.0 Hz, 2H), 7.86 (dd, J = 7.4, 1.0 Hz, 2H), 7.27 (t, J = 7.7 Hz, 2H) ppm. ^{13}C{^{1}H} NMR (151 MHz, $CDCl_3$) δ 147.4, 141.5, 137.1, 131.4, 121.8, 90.4, 31.3 ppm. HRMS (ESI, positive ion mode) m/z: $[M + H]^+$ Calcd for $C_{12}H_7I_2O_2S$ 468.8251; found 468.8248.

Dibenzo[*b*,*d*]thiophene-1,9-dicarboxylic acid 5,5-dioxide (**7**).

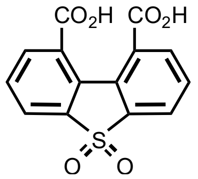

A solution of the dilithio intermediate **2-Li$_2$** was generated as described for **4**. It was cooled to −100 °C and then dry gaseous CO_2 was introduced which was accompanied by a temperature rise to ca. −80 °C. The saturation with CO_2 was continued until the temperature reached 0 °C. The further workup was performed as described for **4**. Yield 0.23 g (76%). ^{1}H NMR (600 MHz, DMSO-d_6) δ 8.19 (dd, J = 7.7, 1.2 Hz, 2H), 8.09 (dd, J = 7.7, 1.2 Hz, 2H), 7.78 (t, J = 7.7 Hz, 2H) ppm. ^{13}C{^{1}H} NMR (151 MHz, DMSO-d_6) δ 167.1, 138.9, 134.8, 132.6, 131.4, 129.7, 124.2 ppm. HRMS (ESI, negative ion mode) m/z: [M − H]$^-$ Calcd for $C_{14}H_7O_6S$ 302.9969; found 302.9968.

9-(9-ethyl-9*H*-carbazol-3-yl)-8*H*-4-thia-9-azacyclohepta[*def*]fluorene-8,10(9*H*)-dione 4,4-dioxide (**8**).

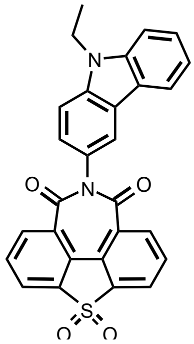

A mixture of **7** (152 mg, 0.5 mmol) and thionyl chloride (2 mL) was refluxed for 4 hrs. The resulting mixture was concentrated to leave a solid which was suspended in dichloromethane (5 mL). A solution of 3-amino-9-ethylcarbazole (105 mg, 0.5 mmol) and triethylamine (0.20 g, 2.0 mmol) in DCM (5 mL) was added and the obtained yellow mixture was stirred for 3 h at rt followed by addition of water (10 mL). DCM was removed under reduced pressure to leave a suspension which was filtered and the collected solid was washed with water (5 mL), Et_2O (5 mL) and dried. Then it was suspended in a mixture of AcOH (0.5 mL) and acetic anhydride (1.0 mL) followed by heating at 140 °C for 1 hr. The mixture was cooled to the room temperature and water (5 mL) was added. The mixture was stirred for 2 hrs and filtered. The collected solid was washed with water (5 mL), Et_2O (5 mL) and dried, to give the product as a pale yellow solid. Yield 153 mg (64%). ^{1}H NMR (300 MHz, DMSO-d_6) δ 8.54 (dd, J = 8.0, 1.1 Hz, 2H), 8.48 (dd, J = 7.6, 1.1 Hz, 2H), 8.09 (dt, J = 7.7, 1.0 Hz, 1H), 8.03 (d, J = 2.2 Hz, 1H), 7.97 (t, J = 7.8 Hz, 2H), 7.71 (d, J = 9.3 Hz, 1H), 7.66 (dt, J = 8.4, 0.9 Hz, 1H), 7.49 (ddd, J = 8.3, 7.1, 1.2 Hz, 1H), 7.37 (dd, J = 8.6, 2.0 Hz, 1H), 7.21 (ddd, J = 7.9, 7.1, 0.9 Hz, 1H), 4.52 (q, J = 7.0 Hz, 2H), 1.39 (t, J = 7.1 Hz, 1H) ppm. ^{13}C{^{1}H} NMR (151 MHz, DMSO-d_6) δ 164.8, 139.9, 138.5, 138.0, 137.1, 132.8, 132.2, 129.6, 128.0, 126.8, 126.0, 125.9, 122.3, 122.0, 120.3, 119.6, 118.9, 109.3, 37.1, 21.1, 13.7 ppm. HRMS (ESI, positive ion mode) m/z: [M]$^{•+}$ Calcd for $C_{28}H_{18}N_2O_4S$ 478.0982; found 478.0981.

Phenanthro[4,5-*bcd*]thiophene-8,9-dione 4,4-dioxide (9).

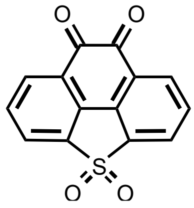

A solution of **2** (0.75 g, 2.0 mmol) in THF (10 mL) was added dropwise to a solution of *n*BuLi (2.5 M in hexane, 1.8 mL, 4.5 mmol) in THF (20 mL) and Et_2O (10 mL) at –90 °C. The olive-brown solution was stirred at –90 °C for ca. 30 min followed by the addition of solid *N*,*N*'-dimethylpiperazine-2,3-dione (0.31 g, 2.2 mmol) as the electrophile. The mixture was allowed to warm to the room temperature and hydrolyzed with aq. 2 M HCl (5 mL). Et_2O (20 mL) was added and the yellow-orange organic phase was separated. Solvents were removed under reduced pressure to leave the solid residue which was washed with Et_2O (2 × 5 mL). It was recrystallized in acetone (3 mL) to give **9** as a yellow powder. Yield 232 mg (43%). ^1H NMR (300 MHz, $CDCl_3$) δ 8.23 (dd, *J* = 7.8, 0.9 Hz, 2H), 8.06 (dd, *J* = 7.7, 0.8 Hz, 2H), 7.75 (t, *J* = 7.8 Hz, 2H) ppm. $^{13}C\{^1H\}$ NMR (151 MHz, $CDCl_3$) δ 176.8, 137.8, 133.2, 132.6, 131.7, 129.1, 128.6 ppm. HRMS (ESI, positive ion mode) m/z: $[M + H]^+$ Calcd for $C_{14}H_7O_4S$ 271.0060; found 271.0061.

Thieno[2',3',4',5':4,5]phenanthro[9,10-*b*]pyrazine-9,10-dicarbonitrile 4,4-dioxide (10).

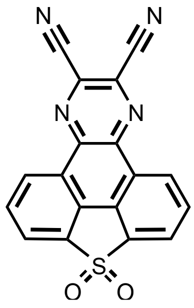

A mixture of **9** (54 mg, 0.2 mmol), diaminomaleonitrile (22 mg, 0.2 mmol) and AcOH (1 mL) was stirred at 110 °C for 2 h. The mixture was cooled to rt and diluted with water (10 mL) followed by extraction with dichloromethane (2 × 20 mL). The collected extract was dried with Na_2SO_4 and filtered through a Celite pad. The filtrate was evaporated to dryness and Et_2O (5 mL) was added. The suspension was filtered to give the product as a beige solid. Yield 25 mg (37%). ^1H NMR (600 MHz, $CDCl_3$) δ 9.07 (dd, *J* = 8.1, 0.7 Hz, 2H), 8.30 (dd, *J* = 7.5, 0.7 Hz, 2H), 8.09 (dd, *J* = 8.1, 7.5 Hz, 2H) ppm. $^{13}C\{^1H\}$ NMR (151 MHz, $CDCl_3$) δ 143.1, 137.4, 131.9, 131.1, 130.3, 128.6, 125.8, 125.6, 113.2 ppm. HRMS (ESI, negative ion mode) m/z: $[M]^{\bullet-}$ Calcd for $C_{18}H_6N_4O_2S$; 342.0217; found 342.0217.

9-(Anthracen-9-yl)-8*H*-thieno[2',3',4',5':4,5]phenanthro[9,10-*d*]imidazole 4,4-dioxide (11).

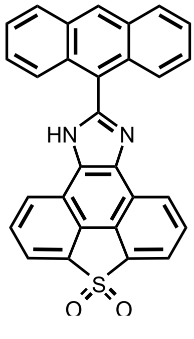

A mixture of **9** (54 mg, 0.2 mmol), 9-anthraldehyde (41 mg), ammonium acetate (500 mg), and AcOH (1 mL) was stirred at 110 °C for 1 h. The mixture was cooled to rt and diluted with water (10 mL). The resulting suspension was filtered and the collected solid was filtered, washed with water and dried. It was dissolved in DMSO (30 mL) and the resulting solution was filtered through a Celite pad. The filtrate was evaporated almost

to dryness and water (5 mL) was added. The suspension was filtered; the collected solid was washed with water and dried, to give the product as a beige solid. Yield 32 mg (35%). ^{1}H NMR (300 MHz, DMSO-d_6) δ 8.94 (s, 1H), 8.67 (d, *J* = 7.9 Hz, 1H), 8.53 (d, *J* = 8.0 Hz, 1H), 8.31–8.18 (m, 4H), 8.05 (dd, *J* = 8.0, 7.4 Hz, 2H), 7.85–7.75 (m, 2H), 7.70–7.50 (m, 4H) ppm. ^{13}C{^{1}H} NMR (151 MHz, DMSO-d_6) δ 147.8, 137.26, 137.06, 136.8, 130.91, 130.69, 130.39, 130.30, 129.2, 128.6, 127.7, 127.2, 125.77, 125.65, 125.57, 125.31, 125.07, 124.95, 122.70, 122.67, 120.5, 118.12, 117.90 ppm. HRMS (ESI, positive ion mode) m/z: $[M + H]^+$ Calcd for $C_{29}H_{17}N_2O_2S$ 457.1005; found 457.1005.

8-Phenyl-8*H*-4-thia-8-phosphacyclopenta[*def*]fluorene 4,4-dioxide (12).

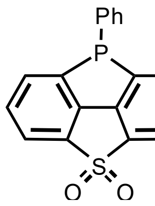

This compound was obtained as described for **9** using a solution of phenyldichlorophosphine (0.30 mL, 2.2 mmol) in Et_2O (5 mL) as the electrophile. The product was purified by recrystallization in a small amount of DCM/Et_2O (1:1, ca. 3 mL): a pale yellow solid was obtained. Yield 0.35 g (54%). ^{1}H NMR (300 MHz, $CDCl_3$) δ 7.84 (d, *J* = 7.4 Hz, 2H), 7.69 (dd, *J* = 7.6, 0.5 Hz, 2H), 7.52 (td, *J* = 7.5, 3.1 Hz, 2H), 7.41–7.27 (m, 5H) ppm. ^{13}C{^{1}H} NMR (151 MHz, $CDCl_3$ δ 140.7 (d, *J* = 24.5 Hz), 139.6 (d, *J* = 4.0 Hz), 137.1, 134.7 (d, *J* = 15.8 Hz), 132.5 (d, *J* = 21.2 Hz), 132.0 (d, *J* = 15.1 Hz), 131.5 (d, *J* = 4.2 Hz), 130.3, 129.2 (d, *J* = 8.0 Hz), 121.8 ppm. ^{31}P NMR (122 MHz, $CDCl_3$) δ 18.0 ppm. HRMS (ESI, positive ion mode) m/z: $[M + H]^+$ Calcd for $C_{18}H_{12}O_2PS$ 323.0290; found 323.0292.

8-Phenyl-4-thia-8-phosphacyclopenta[*def*]fluorene 4,4,8-trioxide (13).

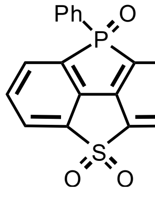

A solution of **12** (161 mg, 0.5 mmol) in dichloromethane (5 mL) was treated with *meta*-chloroperoxybenzoic acid (mCPBA) (70wt%, 150 mg, 0.6 mmol) at rt. The mixture was stirred for 2 h. The hydrolysis was performed using aq. 10 wt% Na_2SO_3 (5 mL) in order to reduce an excess of an electrophile. The reaction mixture was extracted with DCM (20 mL). The organic phase was washed consecutively with aq. NaOH (1 M, 10 mL), water (10 mL) and dried with Na_2SO_4. Solvents were removed in vacuo to leave the solid residue which was suspended in Et_2O (5 mL) and stirred for 30 min. The suspension was filtered to give the product as cream white solid. Yield 151 mg (89%). ^{1}H NMR (600 MHz, $CDCl_3$) δ 7.81 (dd, *J* = 7.3, 6.1 Hz, 2H), 7.79 (dd, *J* = 7.6, 2.0 Hz, 2H), 7.71 (ddd, *J* = 13.7, 8.3, 1.4 Hz, 2H), 7.61–7.56 (m, 3H), 7.48 (td, *J* = 7.8, 3.4 Hz, 2H) ppm. ^{13}C{^{1}H} NMR (151 MHz, $CDCl_3$) δ 137.8 (d, *J* = 23.5 Hz), 136.6 (d, *J* = 9.0 Hz), 134.4 (d, *J* = 9.1 Hz), 133.6, 133.5, 130.83 (d, *J* = 111.4 Hz), 130.79 (d, *J* = 11.8 Hz), 129.4 (d, *J* = 13.3 Hz), 128.9 (d, *J* = 104.0 Hz), 126.4 (d, *J* = 2.4 Hz) ppm. ^{31}P NMR (243

MHz, $CDCl_3$) δ 43.6 ppm. HRMS (ESI, positive ion mode) m/z: $[M + H]^+$ Calcd for $C_{18}H_{12}O_3PS$ 339.0239; found 339.0241.

Single crystal X-ray crystallography

Crystal structure determination. Single crystals suitable for X-ray diffraction measurements were obtained by slow evaporation of $CHCl_3$ (compounds **2**, **12**, **13**) or acetone (**5**) solutions. The crystallizations were performed in open vials at room temperature. X-ray diffraction data were collected on a SuperNova diffractometer (T = 100 K) equipped with Atlas detector using Cu-Kα radiation (λ = 1.54184 Å). Data reduction and analysis were carried out with the CrysAlisPro program.[2] The structures were solved via direct methods using SHELXS-97[3] and refined using SHELXL-2016.[4] All non-hydrogen atoms were refined anisotropically. All C–H hydrogen atoms were placed in calculated positions with C–H distances of 0.95 Å and U_{iso}(H) = 1.2U_{eq}(C). The positions of H atoms of hydroxy groups were located from a difference electron density maps. The O–H distances were fixed to 0.84 Å with a standard deviation of 0.02 Å and the directionality of O–H was refined freely. The U_{iso}(H) parameter was set to 1.5U_{eq} with respect to oxygen atoms. Selected crystal data are summarized in **Table S1**. The CIF files can be retrieved from the Cambridge Structural Database deposition numbers: 2456307 (**2**), 2456308 (**5**), 2456309 (**12**), 2456310 (**13**) or the Supporting Information.

Table S1. Selected crystal data, data collection and refinement parameters for **2**, **5**, **12** and **13**.

	**2**	**5**	**12**	**13**
Empirical formula	$C_{12}H_6O_2SBr_2$	$C_{12}H_9BO_4S$	$C_{18}H_{11}PSO_2$	$C_{18}H_{11}PSO_3$
Formula weight	374.05	260.06	322.30	338.30
T / K	100.00(10)	100.00(10)	100.00(10)	100.00(10)
Crystal system	monoclinic	monoclinic	orthorhombic	monoclinic
Space group	$P2_1/c$	$P2_1/n$	$P2_12_12_1$	$P2_1/c$
a / Å	13.50230(10)	10.3639(4)	5.3681(2)	10.0416(9)
b / Å	9.90880(10)	10.18240(10)	13.6292(4)	15.1659(9)
c / Å	8.83950(10)	10.9338(7)	19.5477(8)	9.7249(6)
α / °	90	90	90	90
β / °	91.8170(10)	101.179(7)	90	103.636(7)
γ / °	90	90	90	90
V / Å^3	1182.06(2)	1131.94(9)	1430.17(9)	1439.26(18)
Z	4	4	4	4
ρ_{calc} / gcm^{-3}	2.102	1.526	1.497	1.561

μ / mm^{-1}	10.240	2.584	3.100	3.165
F(000)	720.0	536.0	664.0	696.0
Crystal size/mm^3	0.301 × 0.139 × 0.079	0.195 × 0.129 × 0.044	0.256 × 0.059 × 0.033	0.437 × 0.112 × 0.058
Radiation	Cu Kα (λ = 1.54184)	Cu Kα (λ = 1.54184)	Cu Kα (λ = 1.54184)	Cu Kα (λ = 1.54184)
2Θ range for data collection/°	6.55 to 155.998	10.766 to 155.892	7.908 to 149.45	9.062 to 140.398
Index ranges	$-12 \le h \le 16$, $-12 \le k \le 12$, $-11 \le l \le 11$	$-12 \le h \le 13$, $-12 \le k \le 12$, $-12 \le l \le 13$	$-6 \le h \le 6$, $-17 \le k \le 16$, $-24 \le l \le 24$	$-12 \le h \le 12$, $-7 \le k \le 18$, $-11 \le l \le 11$
Reflections collected	12091	14963	6937	8257
Independent reflections	2483 [R_{int} = 0.0541, R_{sigma} = 0.0281]	2407 [R_{int} = 0.0395, R_{sigma} = 0.0288]	2620 [R_{int} = 0.0636, R_{sigma} = 0.0550]	2637 [R_{int} = 0.1105, R_{sigma} = 0.0683]
Data/restraints/parameters	2483/0/154	2407/2/169	2620/0/199	2637/0/208
Goodness-of-fit on F^2	1.117	1.075	1.097	1.332
Final R indexes [I>=2σ (I)]	R_1 = 0.0415, wR_2 = 0.1160	R_1 = 0.0347, wR_2 = 0.0971	R_1 = 0.0619, wR_2 = 0.1649	R_1 = 0.0988, wR_2 = 0.2925
Final R indexes [all data]	R_1 = 0.0420, wR_2 = 0.1166	R_1 = 0.0377, wR_2 = 0.0994	R_1 = 0.0700, wR_2 = 0.1727	R_1 = 0.1170, wR_2 = 0.3235
Largest diff. peak/hole / e Å^{-3}	1.44/–0.87	0.38/–0.38	0.63/–0.53	1.32/–1.27

Crystal structure description

Compound **2**. The molecules of **2** assemble through CH…O interactions involving two H atoms at 3 and 4-positions with an O atom of the sulfonyl group in an adjacent molecule (H…O contacts of 2.66 and 2.52 Å, respectively). The molecules are also linked through intermolecular CH…Br interactions (H…Br contact of 2.93 Å). Overall both types of interactions result in the formation of a layer parallel to the (1 0 −1) plane (**Figure S1**).

Compound **5**. In the molecule of **5**, the aromatic scaffold is only slightly puckered which can be caused by steric hindrance of the boronic group as there is a rather short intramolecular contact of 2.616 Å between the B atom and the H atom in the 9-position. The $B(OH)_2$ group is

strongly twisted with respect to the DBTS core: the dihedral angle between the mean planes of boronic group and the adjacent benzene ring is 63.3°. The molecules assemble through mutual H-bonding interactions of boronic groups giving rise to centrosymmetric dimeric motifs (O…O contact of 2.732(2) Å, **Figure S2**). Apart from these classical "head-to-head" dimers, "head-to-tail" heterodimers can be distinguished due to H-bonding interactions of $B(OH)_2$ and SO_2 groups (O…O distance of 2.735(2) Å). They are strengthened through π-stacking interactions of aromatic scaffolds. Overall, combinations of both types of dimeric motifs produces chains which propagate along the *c* axis.

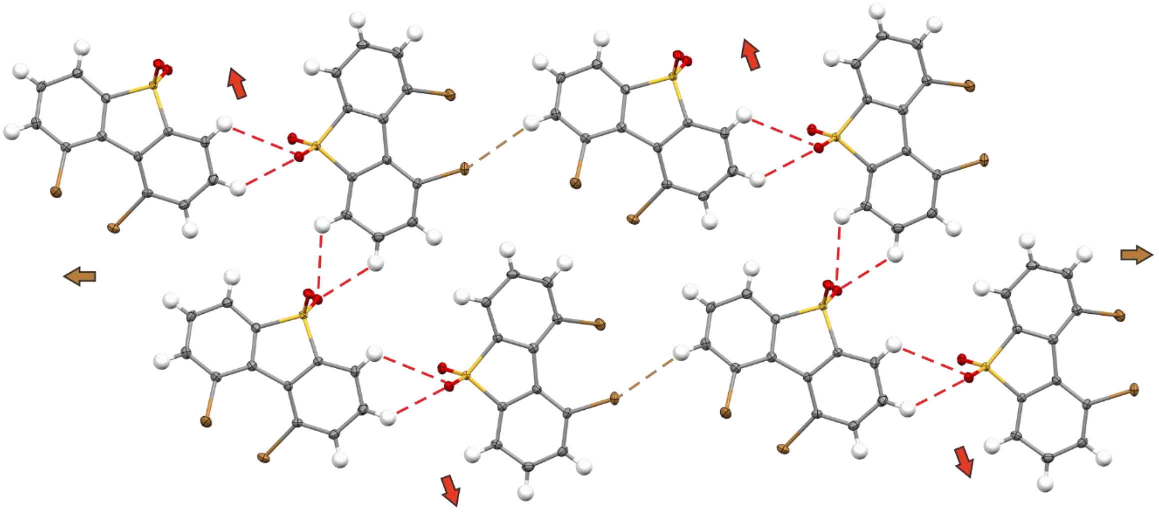

Figure S1. Supramolecular assembly of **2.**

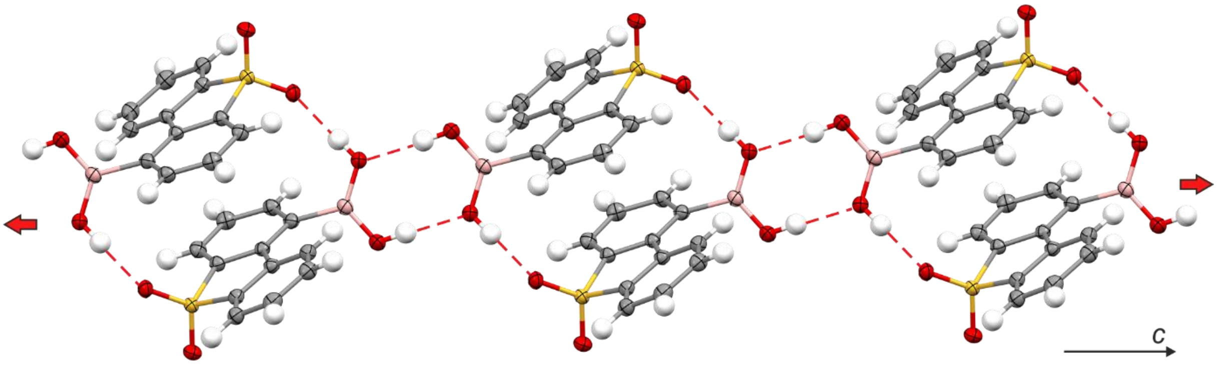

Figure S2. Hydrogen bonded supramolecular assembly of **5.**

Theoretical calculations

Theoretical calculations were performed using *Gaussian16* program.[5] Molecules were optimized using B3LYP (DFT)[6] method with 6-311++G(d,p) basis set.[7] The starting geometries were adopted from corresponding crystal structures or manually modified in the *GaussView* program.[8] Following geometry optimization, the vibrational frequencies were calculated and the results showed that optimized structures (**Figure S3–S4**), are stable geometric structures (no imaginary frequencies, **Table S2**). In optimization processes no symmetry constraints were applied. To optimize the structures of transition states (**2**, **3**, **6** and **7**, see **Figure S5**), the synchronous transit-guided quasi-Newton approach (QST3) was applied. In this method, three input structures are needed: one corresponds to reactants, one to products and one is a guess of a transition state. To verify the structures of the transition states, frequency calculations were carried out at the same level of theory. One imaginary frequency was found in all cases.

Table S2. Computed total energy values and number of imaginary frequencies for the optimized structures.

compound	*E* / a.u.	N. im. Freq.	compound	*E* / a.u.	N. im. Freq.
**DBTS**	−1010.85006	0	**6-TS**	−1032.18194	1
**2**	−6157.90563	0	**7**	−1388.09654	0
**2-TS**	−6157.89571	1	**7-TS**	−1388.08487	1
**2Li2THF**	−1954.87242	0	**8**	−1886.78497	0
**3**	−1625.40733	0	**9**	−1236.35405	0
**3-TS**	−1625.39411	1	**10**	−1457.36737	0
**4**	−1124.19667	1	**11**	−1773.20895	0
**5**	−1186.92703	0	**12**	−1582.70682	0
**6**	−1032.36941	0	**13**	−1657.97352	0

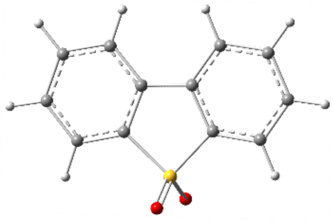

DBTS

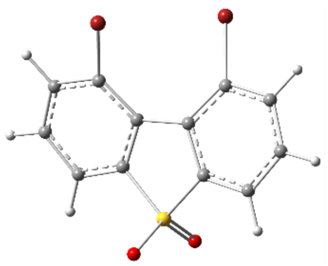

2

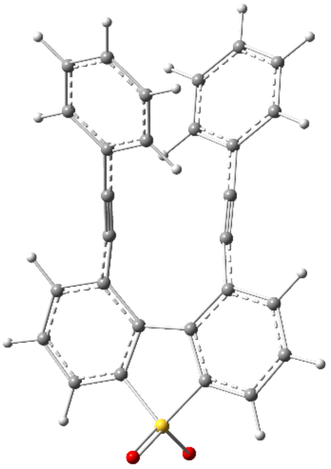

3

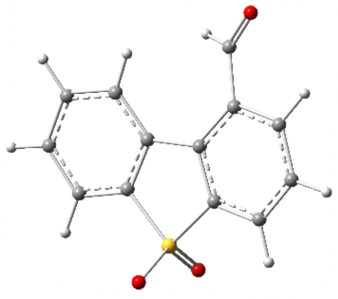

4

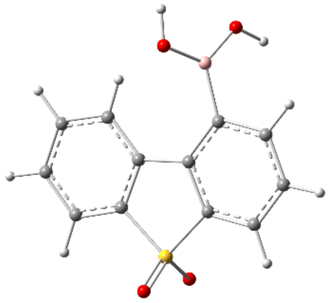

5

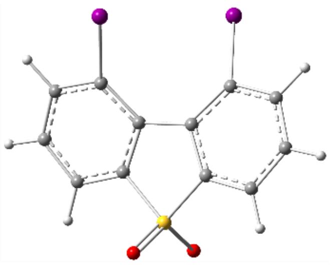

6

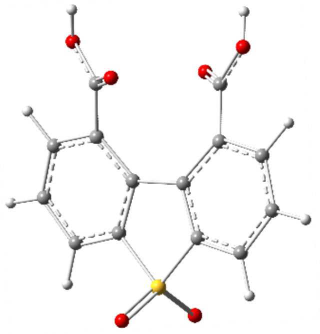

7

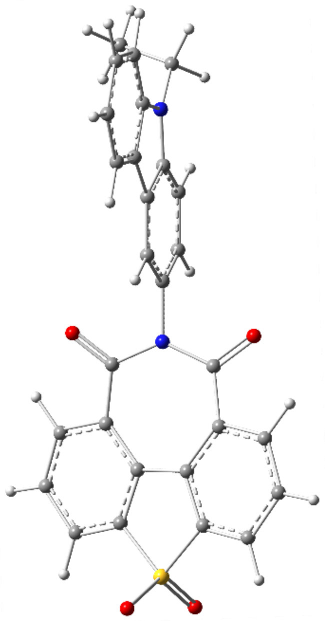

8

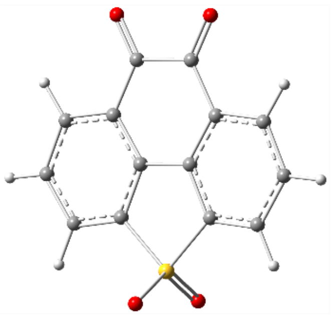

9

Figure S3. The optimized molecular geometries of **DBTS** and **2–9**.

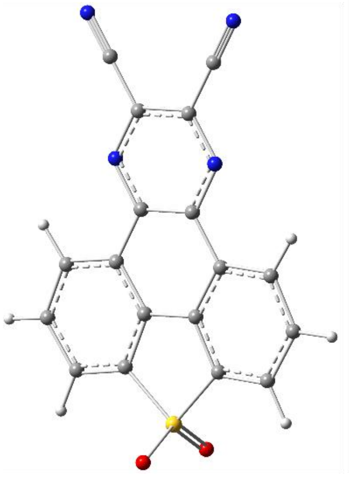

10

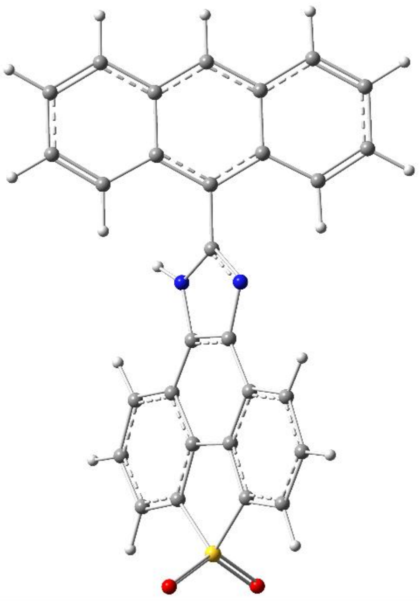

11

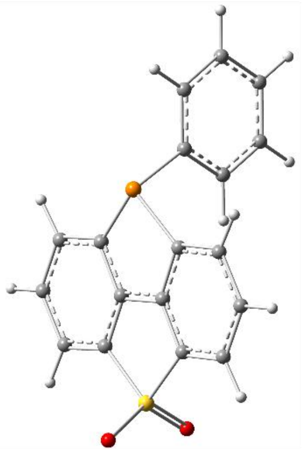

12

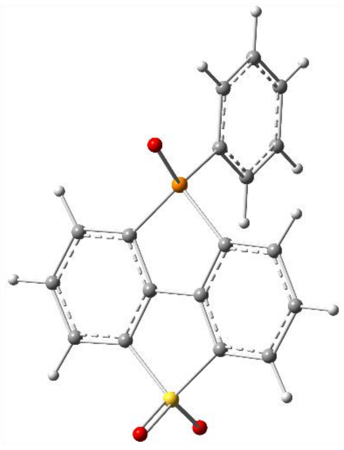

13

Figure S4. The optimized molecular geometries of **10−13**.

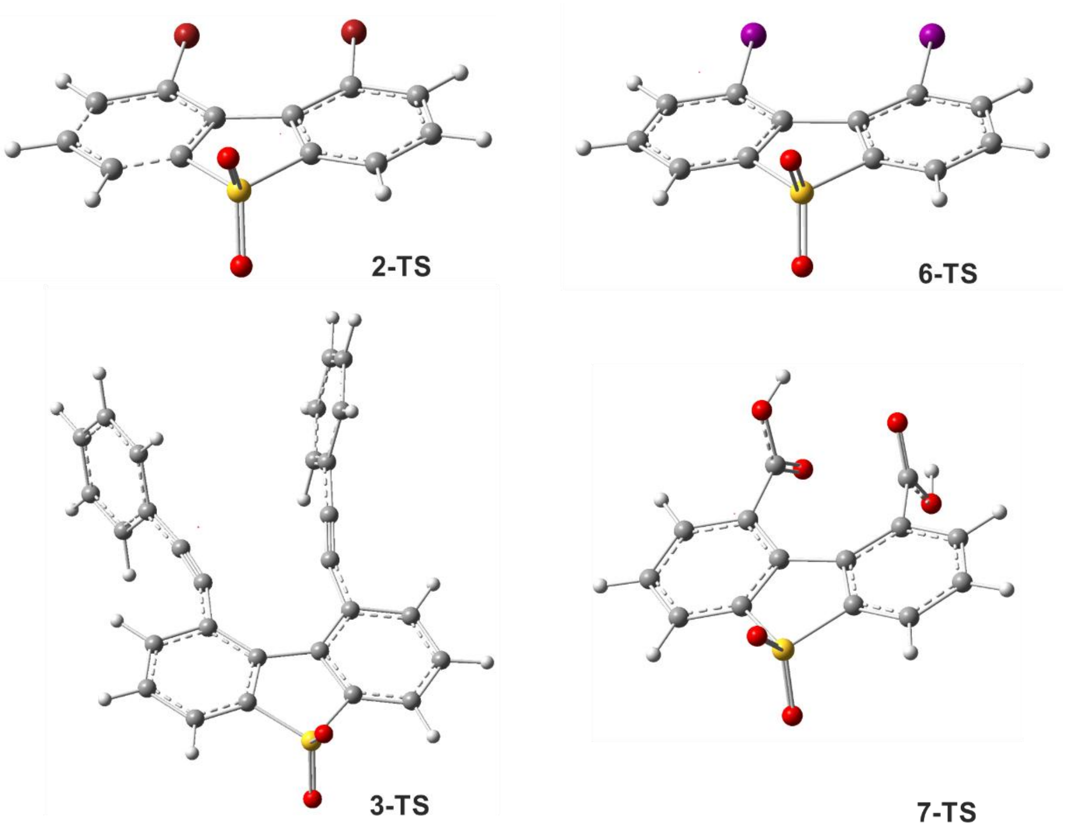

Figure S5. The optimized geometries of transition state structures **2-TS**, **3-TS**, **6-TS** and **7-TS**.

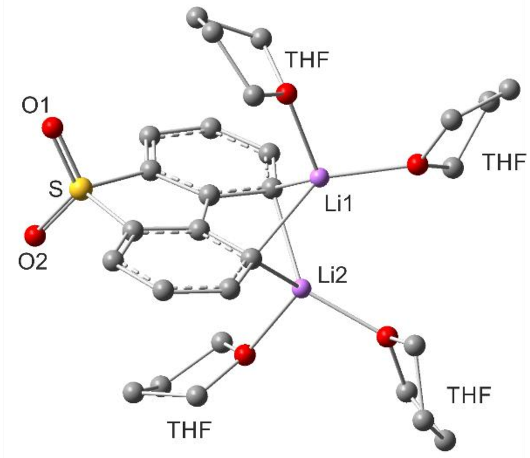

Figure S6. The optimized structure of **2-Li$_2$(THF)$_4$**.

Table S3. Values of τ torsion angles defined by four carbon atoms at the bay positions of DBTS, derived from computed and X-ray experimental geometries.

	τ(DFT)	τ (X-ray)
**DBTS**	0.0	-
**2**	31.4	31.8
**3**	24.7	-
**4**	8.2	-
**5**	1.8	10.2
**6**	35.0	-
**7**	19.5	-
**8**	11.4	-
**9**	0.0	-
**10**	0.0	-
**11**	0.1	-
**12**	0.0	0.6
**13**	0.0	0.2

Table S4. Inversion barriers (E_i) of **2**, **3**, **6** and **7**.and their energies (ΔE) with respect to 3,7-disubstituted isomers.

	**2**	**3**	**6**	**7**
E_i / kJ·mol^{-1}	26	35	46	31
ΔE / kJ·mol^{-1}	64	33	75	58

Table S5. HOMO and LUMO energy levels and energy gaps for **2–13** based on theoretical calculations at the B3LYP/6-311++G(d,p) level of theory.

compound	E_{HOMO} / eV	E_{LUMO} / eV	ΔE^{DFT} / eV
**DBTS**	−7.02	−2.22	4.80
**2**	−7.23	−2.59	4.64
**3**	−6.28	−2.53	3.75
**4**	−7.37	−2.94	4.43
**5**	−6.99	−2.23	4.76
**6**	−6.92	−2.58	4.34
**7**	−7.29	−2.91	4.38
**8**	−5.84	−2.75	3.09
**9**	−5.82	−3.95	3.61
**10**	−7.70	−3.75	3.95
**11**	−5.88	−2.50	3.08
**12**	−6.85	−2.11	4.73
**13**	−7.11	−2.48	4.63

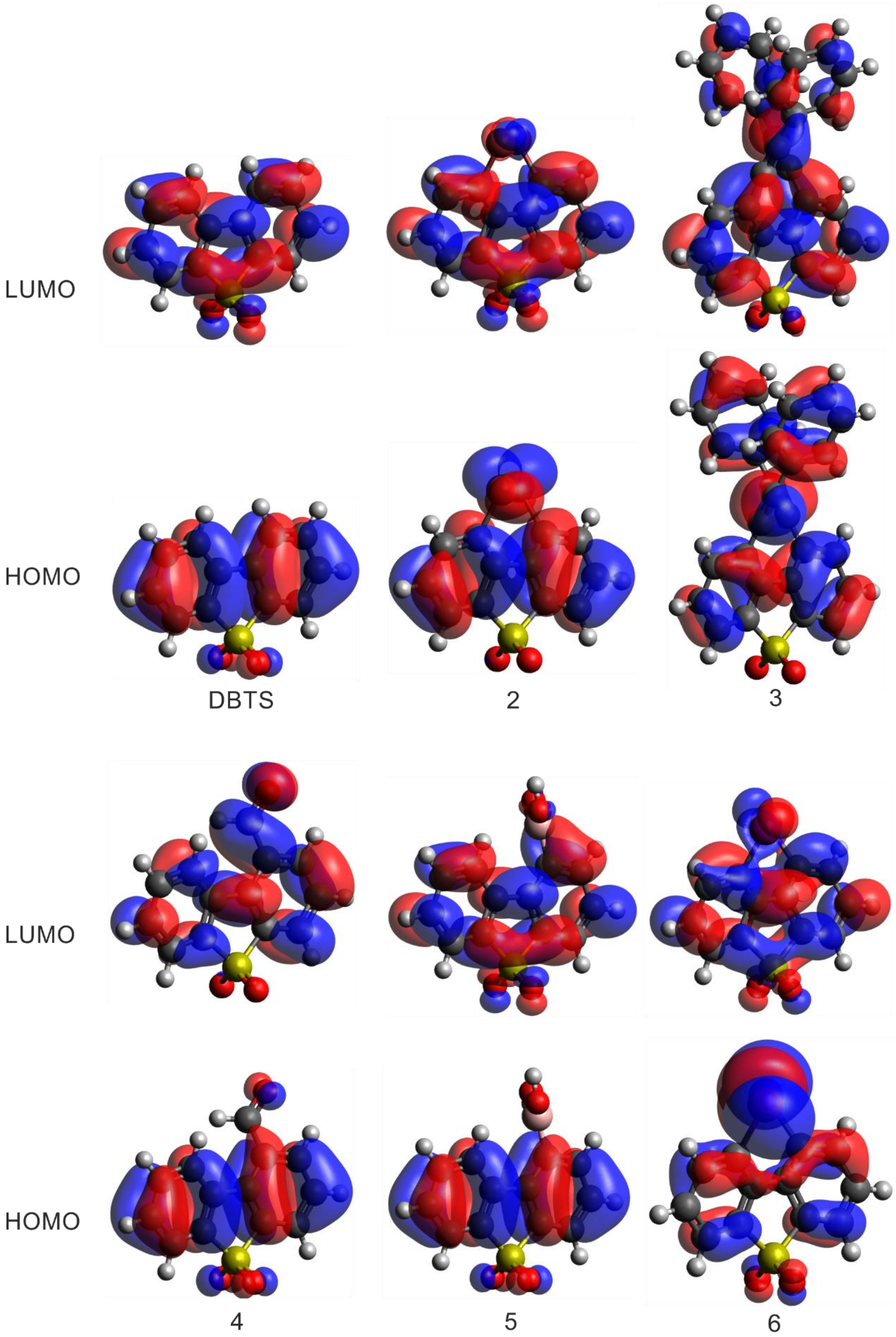

Figure S7. Distribution of frontiers molecular orbitals in **DBTS** and **2–6**. Calculated at B3LYP/6-311++G(d,p) level of theory.

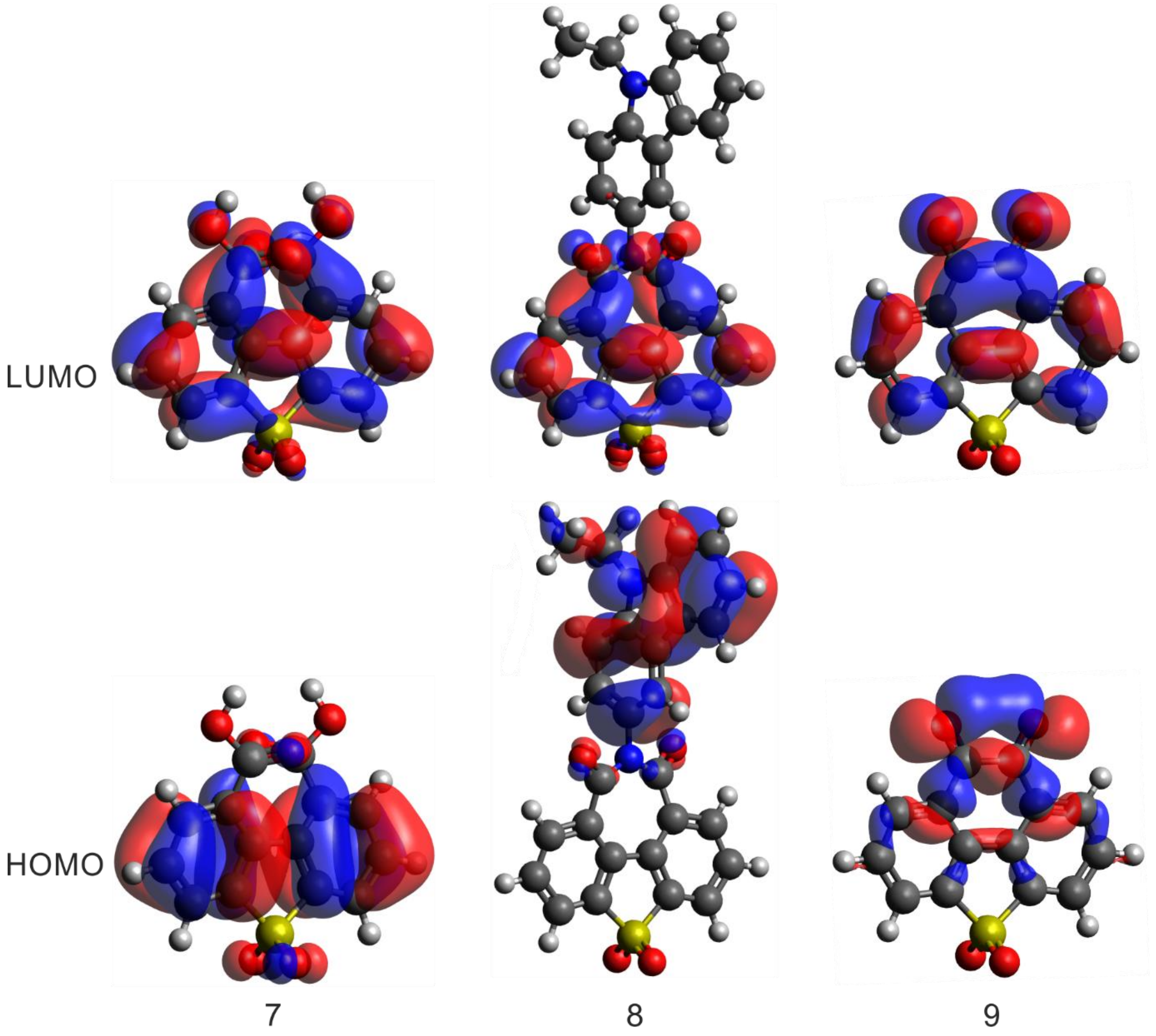

Figure S8. Distribution of frontiers molecular orbitals in **7–9**. Calculated at B3LYP/6-311++G(d,p) level of theory.

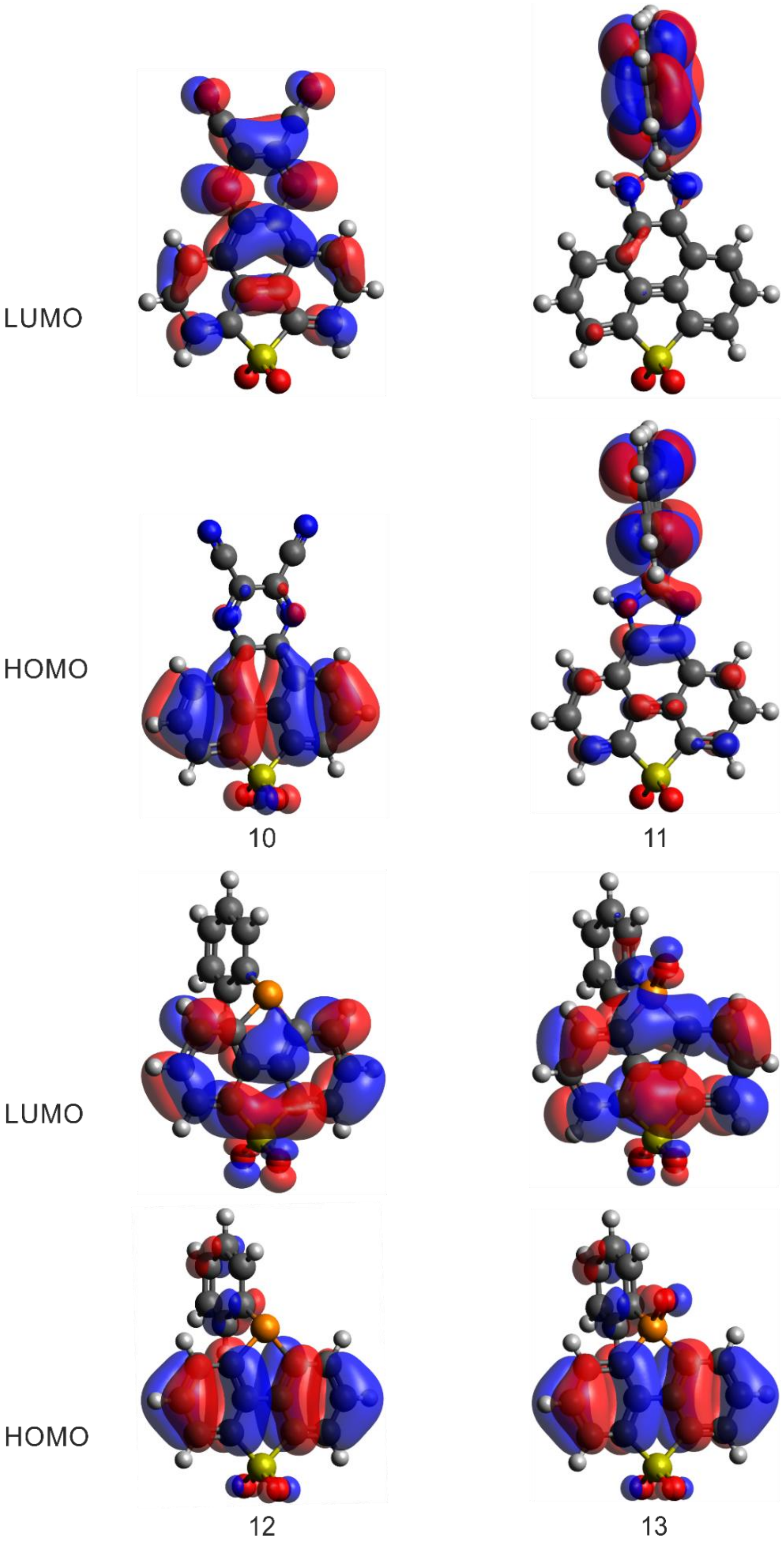

Figure S9. Distribution of frontiers molecular orbitals in **10–13**. Calculated at B3LYP/6-311++G(d,p) level of theory.

Electrochemistry

Cyclic voltammetry was conducted in a three-electrode, one-compartment cell using Ossila Potentiostat (Ossila BV) controlled by Ossila Electrochemistry software (version 1.5). All measurements were performed using 0.1 M Bu_4NBF_4 (99%, Sigma Aldrich, dried) solution in MeCN (compounds **2**–**7**, **9**–**10**, **12**–**13**) or DMSO (compounds **8** and **11**). Solvents were distilled over CaH_2 and stored under argon atmosphere over molecular sieves 4 Å in Rotaflo Schlenk tube at RT. All solutions were bubbled with argon prior to measurement and the measurement was conducted in argon atmosphere (argon inlet was maintained over the solution during the experiments). Electrodes: working (glassy carbon disk electrode, GCE from Mineral, Poland, area = 0.07 cm^2), counter (Pt wire), reference (Ag/AgCl calibrated against ferrocene). Prior each measurement, the working electrode was polished using 1 μm, 0.3 μm and 0.05 μm Al_2O_3 powders, each deposited on separate polishing pads (in form of water slurry) according to the described procedure (the infinite-type motions were applied).[9] All cyclic voltammetry measurements were performed at room temperature with a scan rate of 50 $mV \cdot s^{-1}$. The ionization potential (IP) and electron affinity (EA) are obtained from onset redox potentials. Except derivative **11**, the oxidation processes were not observed within potential window. For compound **8**, the oxidation due to the presence of 9-ethylcarbazole-3-yl moiety could not be observed due to the use DMSO as the solvent (this compound is insoluble in MeCN).

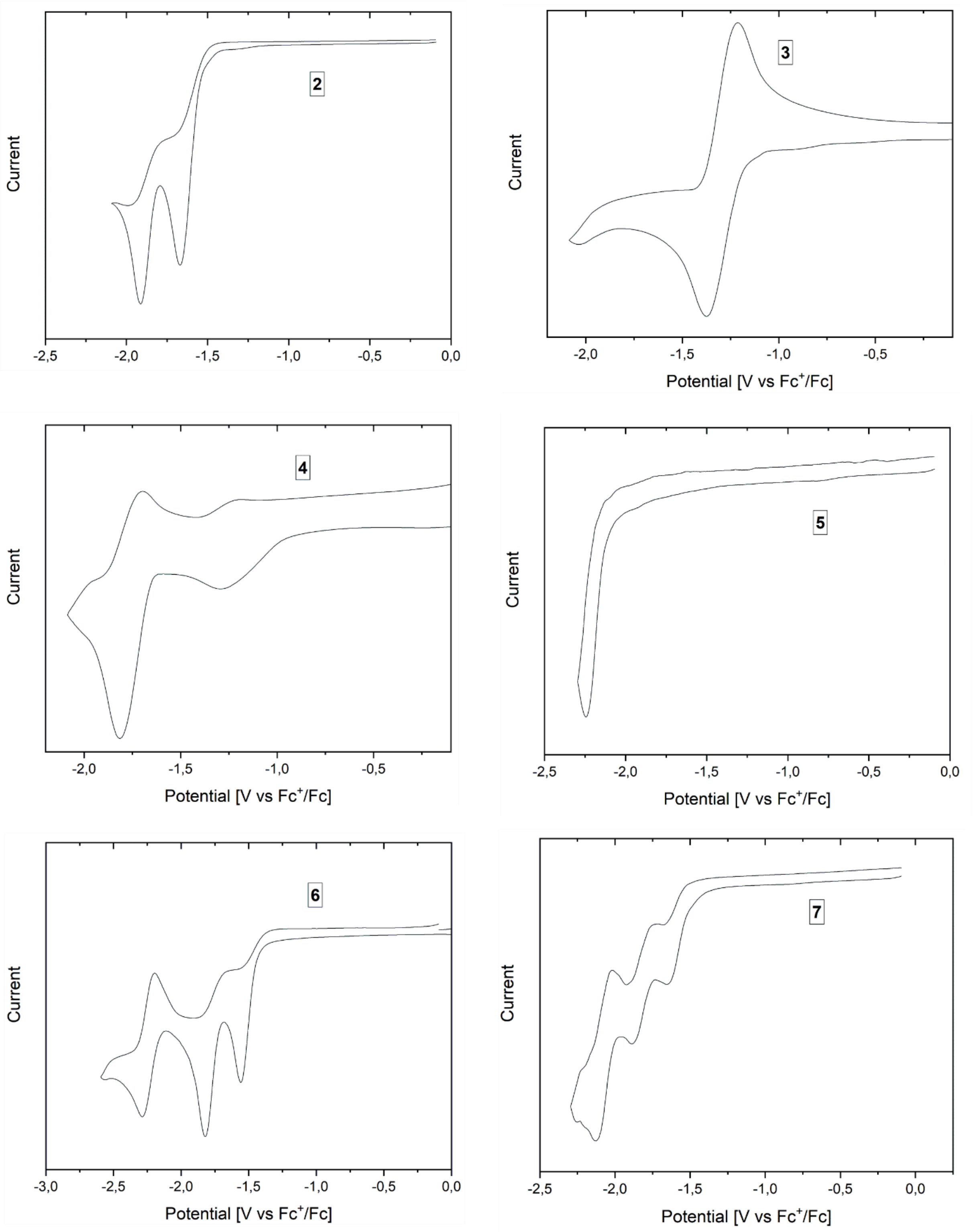

Figure S10. Cyclic voltammograms for compounds **2**–**7**.

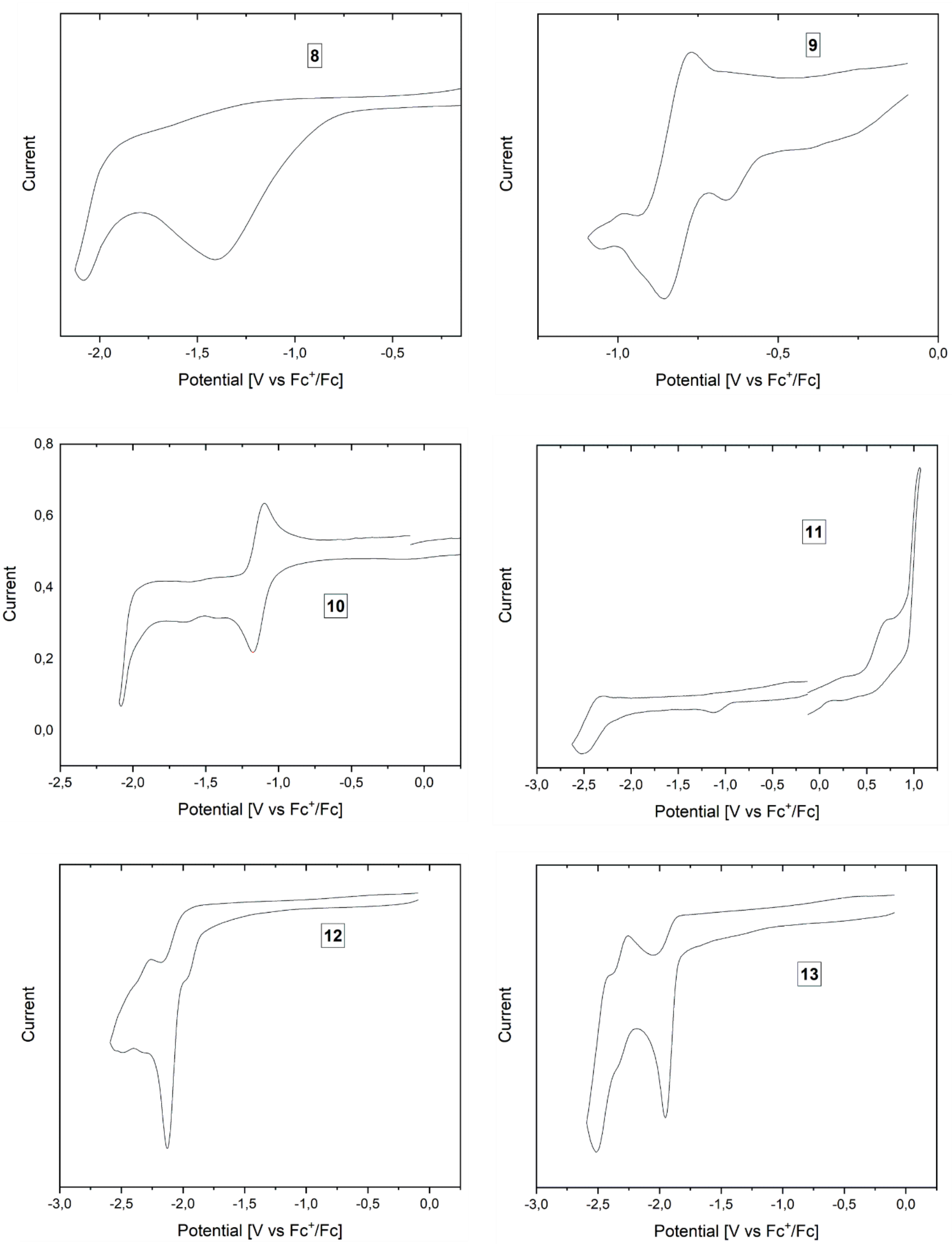

Figure S11. CV for **8-13**.

Table S6. Electrochemical properties of compounds **2–13**.

	E_{red}, eV[a]	E_{ox}, eV[a]	EA, eV [b]	IP, eV [b]
**2**	−1.59	-	3.21	-
**3**	−1.29 (−1.30[c])	1.16	3.51 (3.50)	5.96
**4**	−1.71	-	3.09	-
**5**	−2.11	-	2.69	-
**6**	−1.51	-	3.29	-
**7**	−1.58	-	3.22	-
**8**	−1.21	-	3.59	-
**9**	−0.61	-	4.19	-
**10**	−1.11 (−1.14 [c])	-	3.69 (3.66)	-
**11**	−2.35	0.59	2.45	5.39
**12**	−2.09	-	2.71	-
**13**	−1.90	-	2.90	-

[a] Measured in nBu$_4$NPF$_6$ solution vs. Ag/AgCl (calibration using the FeCp$_2$/FeCp$_2^+$ redox pair using ferrocene) and estimated as the inflection-point potential.[10] [b] Calculated from EA/IP = $E_{red/ox}$ +4.8. [c]determined as $E_{½}$ for a reversible process.

UV-Vis spectroscopy

Absorption spectra were recorded using EVOLUTION One Plus UV-Vis spectrophotometer (Thermo Fisher Scientific). Emission and fluorescence quantum yields were recorded using Edinburgh Instruments FS5 fluorescence spectrophotometer. Suprasil quartz cuvettes (10.00 mm) were used. Absorbance and photoluminescence measurements were performed in dilute (10^{-5} M) solutions. All the experiments were conducted at room temperature. Fluorescence decays were recorded with the picosecond pulsed EPLED diode laser (λ_{ex} = 280 nm) as an excitation source using a time-correlated single photon counting technique. Fluorescence quantum yields were determined applying the relative method.[11] 9,10-Diphenylanthracene (cyclohexane, QY^F = 0.97) was used as the QY^F standard.[12] The following formula was applied in order to calculate fluorescence quantum yields of the samples:

$$QY_x^F = QY_r^F \cdot \frac{F_x}{F_r} \cdot \frac{1 - 10^{-A_r}}{1 - 10^{-A_x}} \cdot \frac{n_x^2}{n_r^2}$$

where:

F is the relative integrated photon flux of sample (**x**) and reference (**r**), **A** is the absorbance at the excitation wavelength, and **n** is the refractive index of used solvents.

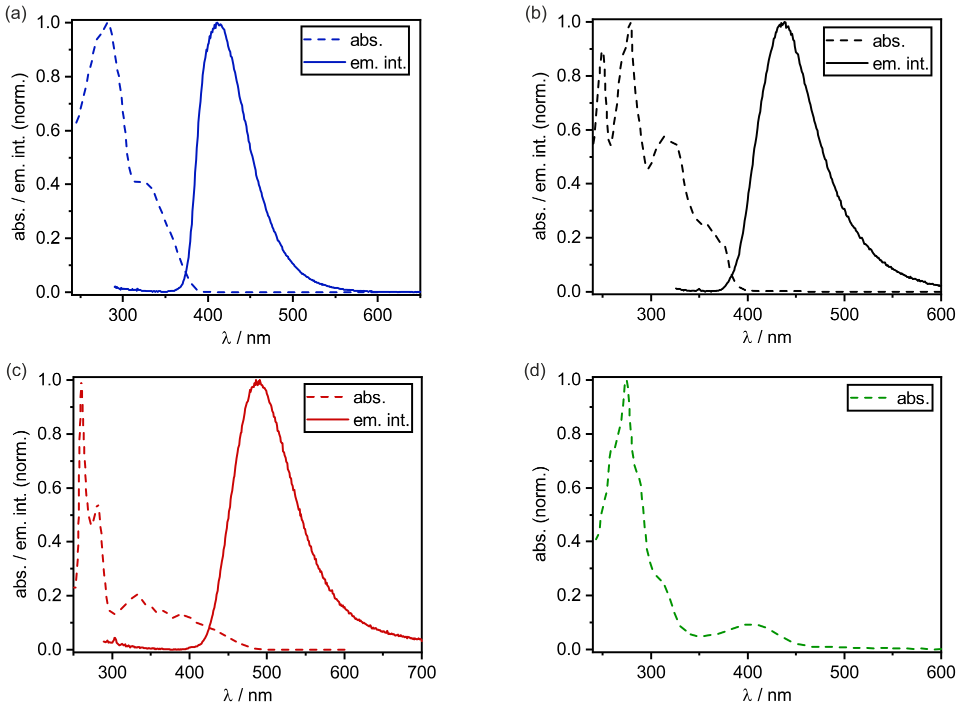

Figure S12. Normalized absorption and emission spectra of compounds (a) **3**, (b) **10**, and (c) **11**. (d) Normalized absorption spectra of compound **9** (emission spectra was not recorded due to very weak fluorescence).

References

(1) Hu, Y.; Wang, Z.; Jiang, X.; Cai, X.; Su, S.-J.; Huang, F.; Cao, Y. One-step synthesis of cyclic compounds towards easy room-temperature phosphorescence and deep blue thermally activated delayed fluorescence. *Chem. Commun.* **2018**, *54*, 7850—7853.

(2) Rigaku Oxfrod Diffraction, CrysAlis PRO 1.171. 38.43, 2015.

(3) Sheldrick, G. M. A Short History of *SHELX. Acta Crystallogr. A* **2008**, *64*, 112–122.

(4) Sheldrick, G. M. Crystal Structure Refinement with *SHELXL*. *Acta Crystallogr. C* **2015**, *71*, 3–8.

(5) M. J. Frisch, G. W. Trucks, H. B. Schlegel, G. E. Scuseria, M. A. Robb, J. R. Cheeseman, G. Scalmani, V. Barone, G. A. Petersson, H. Nakatsuji, X. Li, M. Caricato, A. V. Marenich, J. Bloino, B. G. Janesko, R. Gomperts, B. Mennucci, H. P. Hratchian, J. V. Ortiz, A. F. Izmaylov, J. L. Sonnenberg, D. Williams-Young, F. Ding, F. Lipparini, F. Egidi, J. Goings, B. Peng, A. Petrone, T. Henderson, D. Ranasinghe, V. G. Zakrzewski, J. Gao, N. Rega, G. Zheng, W. Liang, M. Hada, M. Ehara, K. Toyota, R. Fukuda, J. Hasegawa, M. Ishida, T. Nakajima, Y. Honda, O. Kitao, H. Nakai, T. Vreven, K. Throssell, J. A. Montgomery, Jr., J. E. Peralta, F. Ogliaro, M. J. Bearpark, J. J. Heyd, E. N. Brothers, K. N. Kudin, V. N. Staroverov, T. A. Keith, R. Kobayashi, J. Normand, K. Raghavachari, A. P. Rendell, J. C. Burant, S. S. Iyengar, J. Tomasi, M. Cossi, J. M. Millam, M. Klene, C. Adamo, R. Cammi, J. W. Ochterski, R. L. Martin, K. Morokuma, O. Farkas, J. B. Foresman, and D. J. Fox, Gaussian 16, Revision C.01, Gaussian, Inc., Wallingford CT, 2016.

(6) (a) Becke, A.D. Density-functional thermochemistry. III. The role of exact exchange. *J. Chem. Phys.* **1993**, *98*, 5648−5652. (b) Lee, C.; Yang, W.; Parr, R. G. Development of the Colle-Salvetti correlation-energy formula into a functional of the electron density. *Phys. Rev. B* **1988**, *37*, 785−789. (c) Vosko, S.H.; Wilk, L.; Nusair, M. Accurate spin-dependent electron liquid correlation energies for local spin density calculations: a critical analysis. *Can. J. Phys*. **1980,** *58*, 1200−1211. (d) Stephens, P. J.; Devlin, F. J.; Chabalowski, C. F.; Frisch, M. J. Ab Initio Calculation of Vibrational Absorption and Circular Dichroism Spectra UsingDensity Functional Force Fields. *J. Phys. Chem*. **1994**, *98*, 11623−11627.

(7) Lee, C.; Yang, W.; Parr, R. G. *Phys. Rev. B* **1988**, *37*, 785–789.

(8) GaussView, Version 6.1, Roy Dennington, Todd A. Keith, and John M. Millam, Semichem Inc., Shawnee Mission, KS, 2016.

(9) Elgrishi, N.; Rountree, K. J.; McCarthy, B. D.; Rountree, E. S.; Eisenhart, T. T.; Dempsey, J. L. A Practical Beginner's Guide to Cyclic Voltammetry. *J. Chem. Educ.* **2018**, *95*, 197–206.

(10) Espinoza, E. M.; Clark, J. A., Soliman, J.; Derr, J. B.; Morales, M.; Vullev, V. I. Practical Aspects of Cyclic Voltammetry: How to Estimate Reduction Potentials When Irreversibility Prevails. *J. Electrochem. Soc.* **2019**, *166*, 3175−3187.

(11) Würth, C.; Grabolle, M.; Pauli, J.; Spieles, M.; Resch-Genger, U. Relative and absolute determination of fluorescence quantum yields of transparent samples. *Nat. Protoc.* **2013**, *8*, 1535–1550.

(12) Brouwer, A. M. Standards for photoluminescence quantum yield measurements in solution. *Pure Appl. Chem.* **2011**, *83*, 2213−2228.

NMR spectra

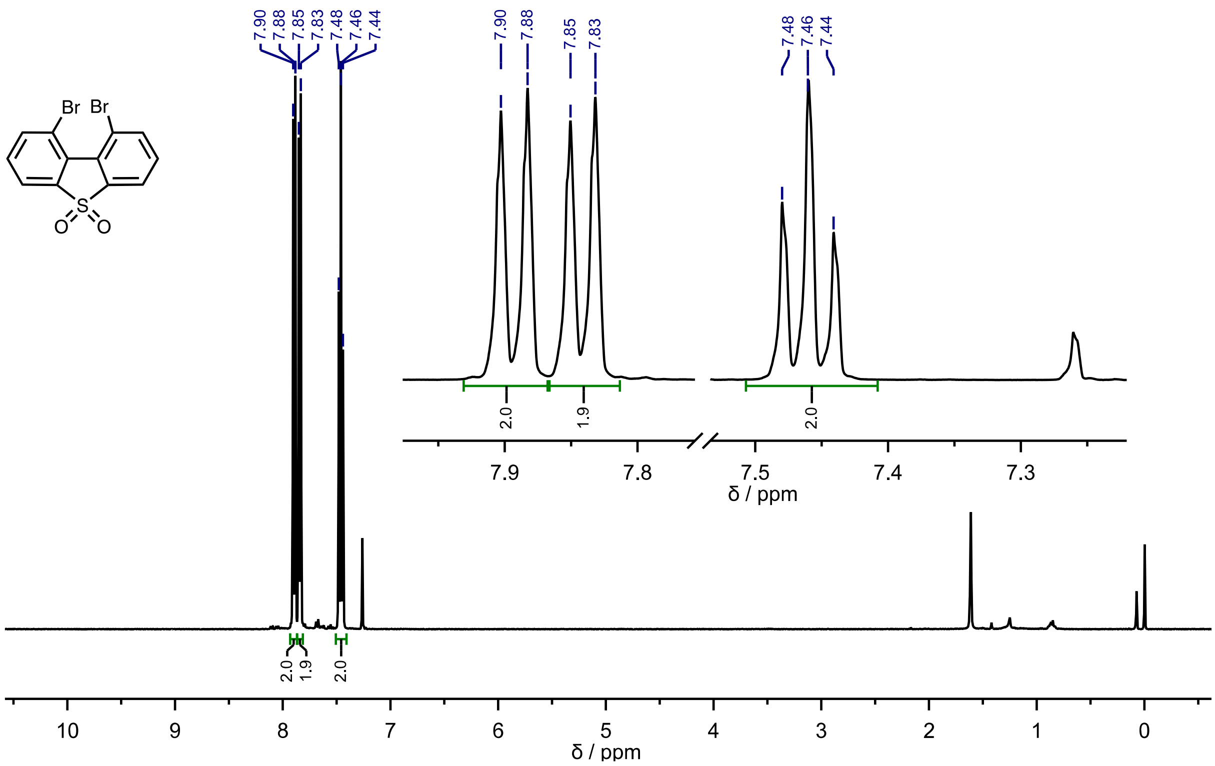

Figure S13. ^{1}H NMR spectrum (600 MHz, $CDCl_3$) of compound **2**.

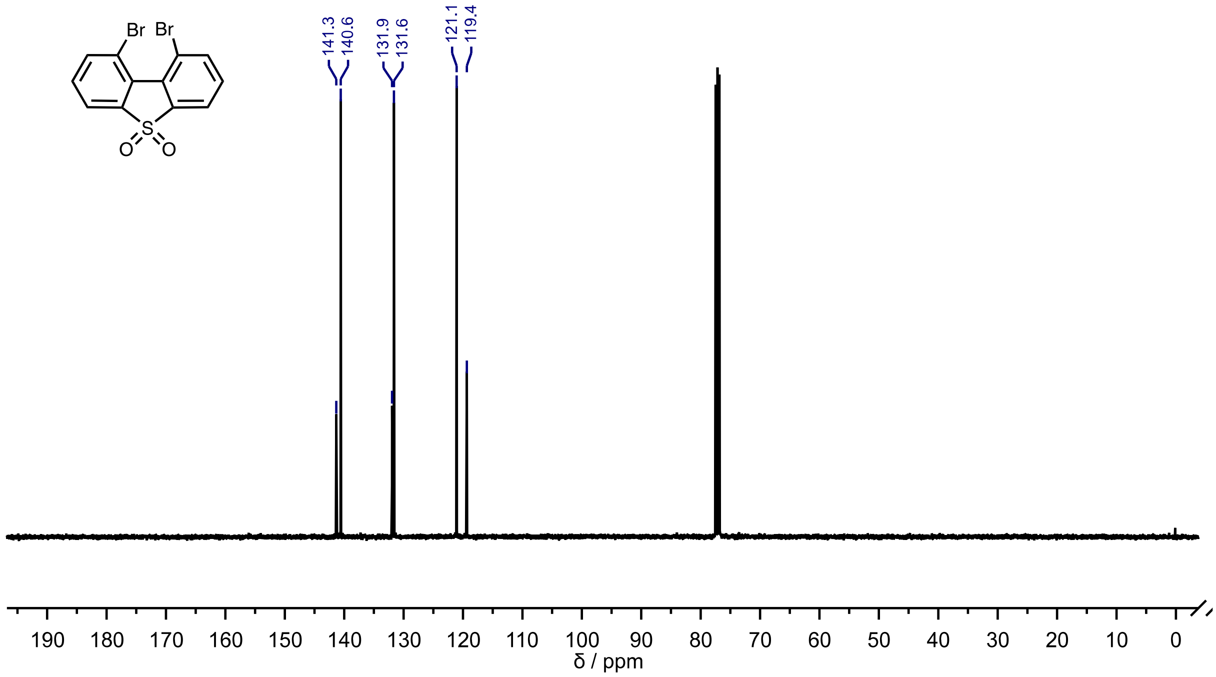

Figure S14. $^{13}C\{^1H\}$ NMR spectrum (151 MHz, $CDCl_3$) of compound **2**.

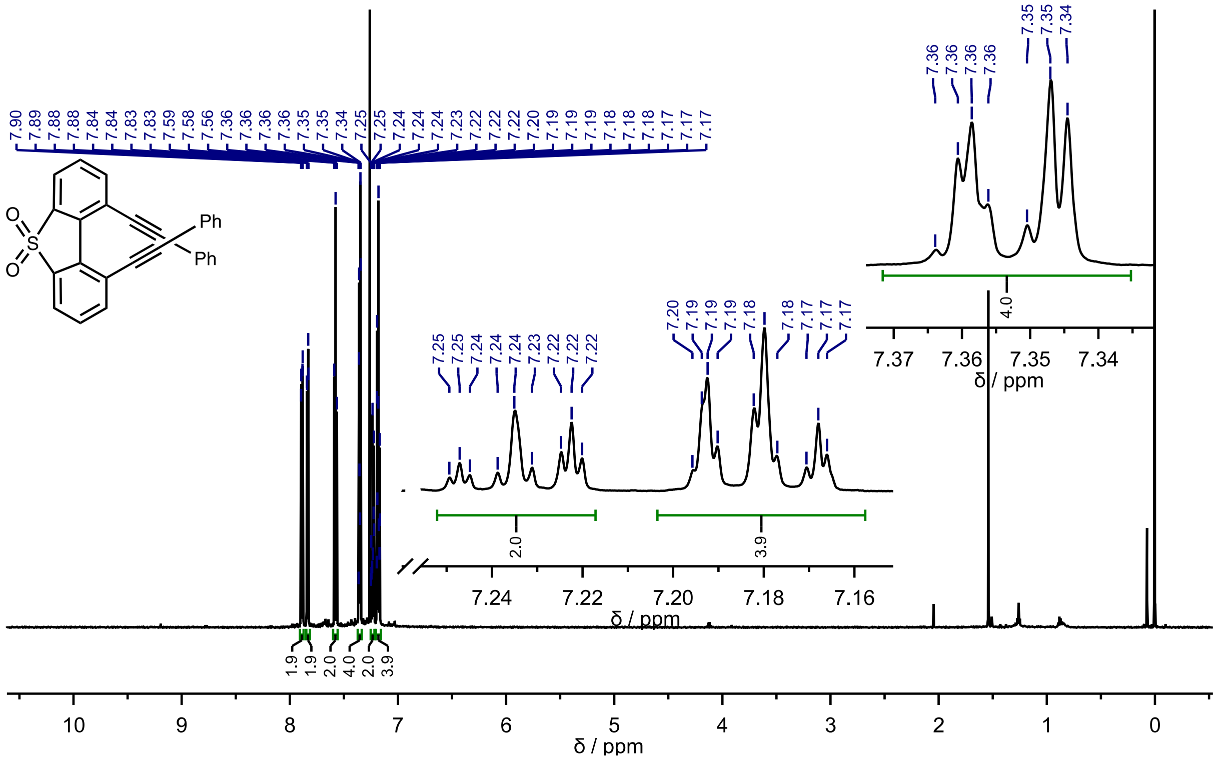

Figure S15. ^{1}H NMR spectrum (600 MHz, $CDCl_3$) of compound **3**.

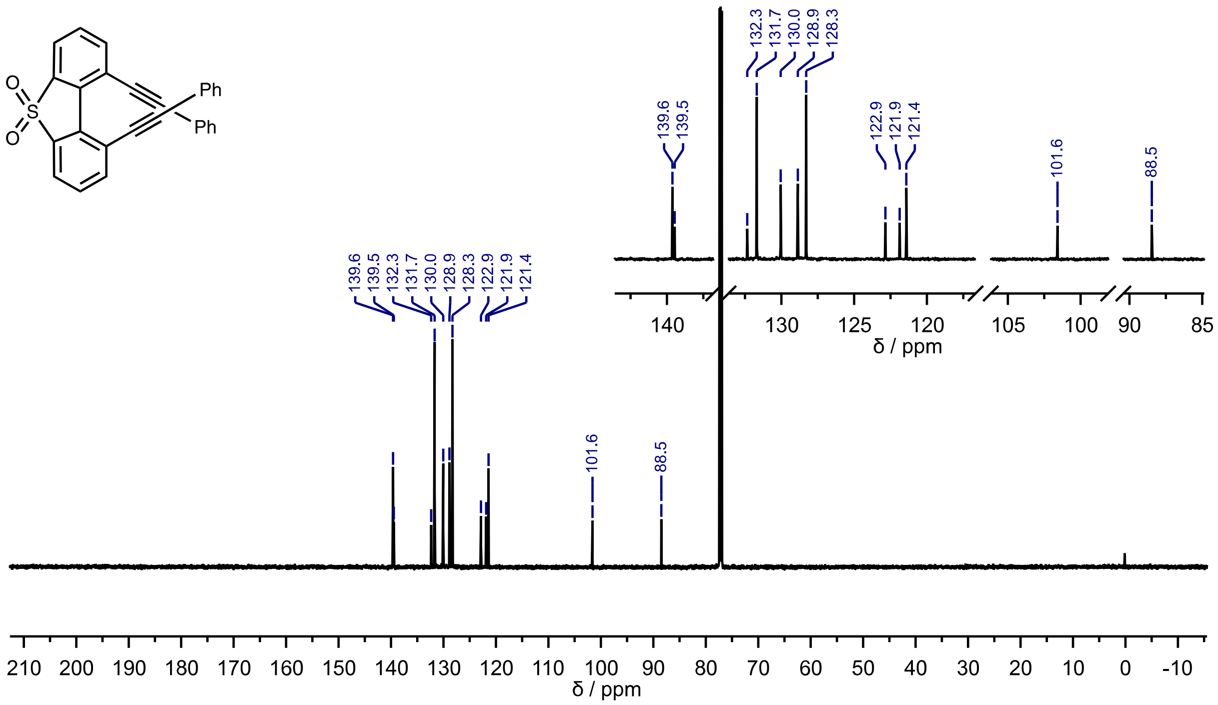

Figure S16. $^{13}C\{^{1}H\}$ NMR spectrum (151 MHz, $CDCl_3$) of compound **3**.

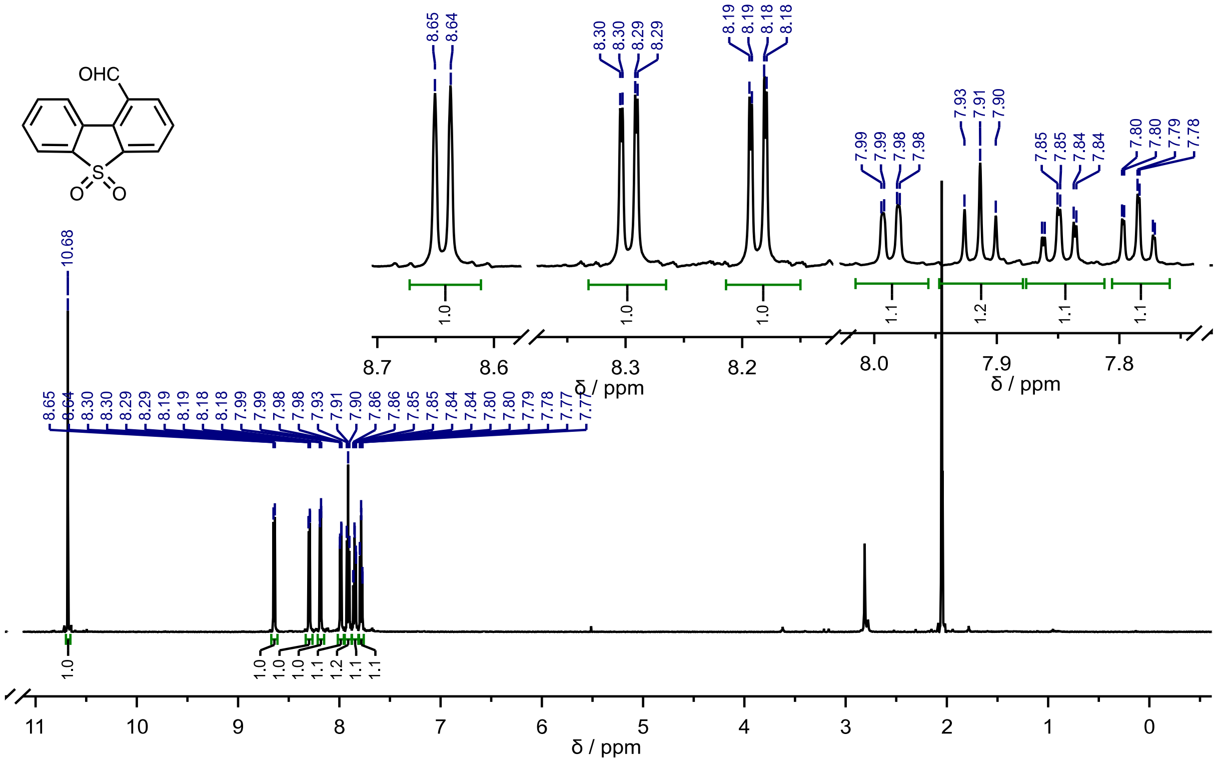

Figure S17. ^{1}H NMR spectrum (600 MHz, acetone-d_6) of compound **4**.

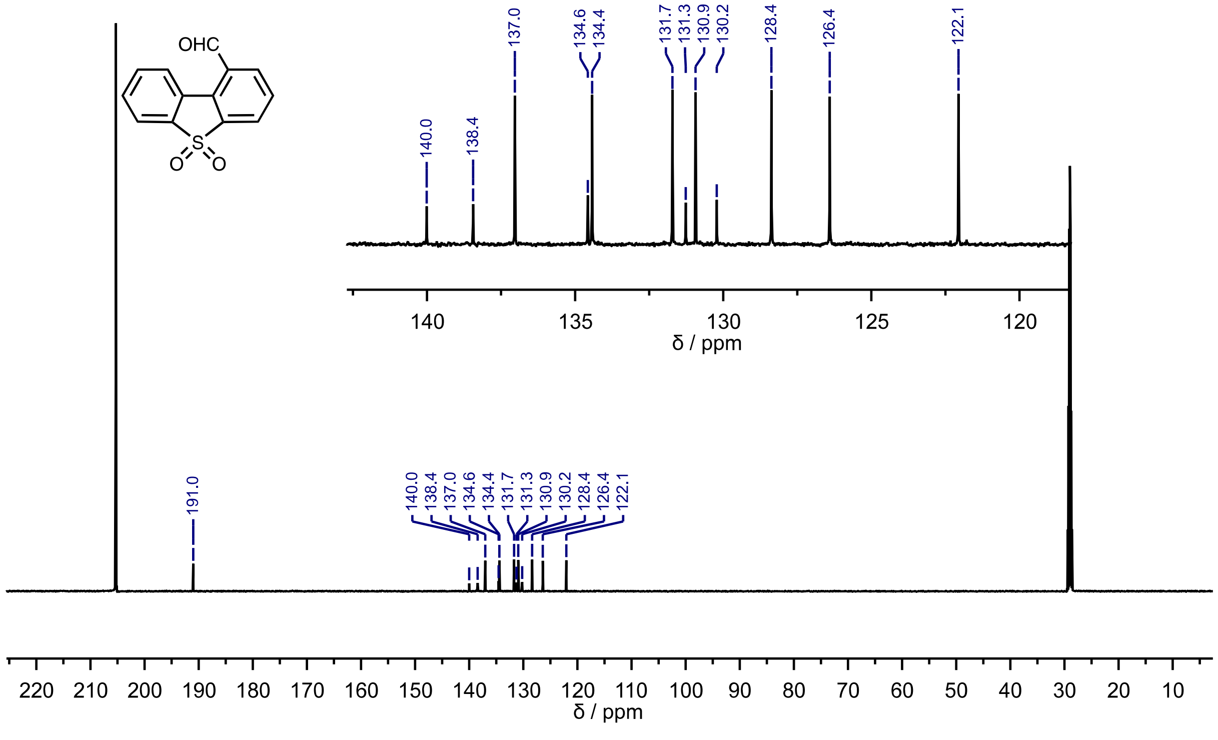

Figure S18. ^{13}C{^{1}H} NMR spectrum (151 MHz, acetone-d_6) of compound **4**.

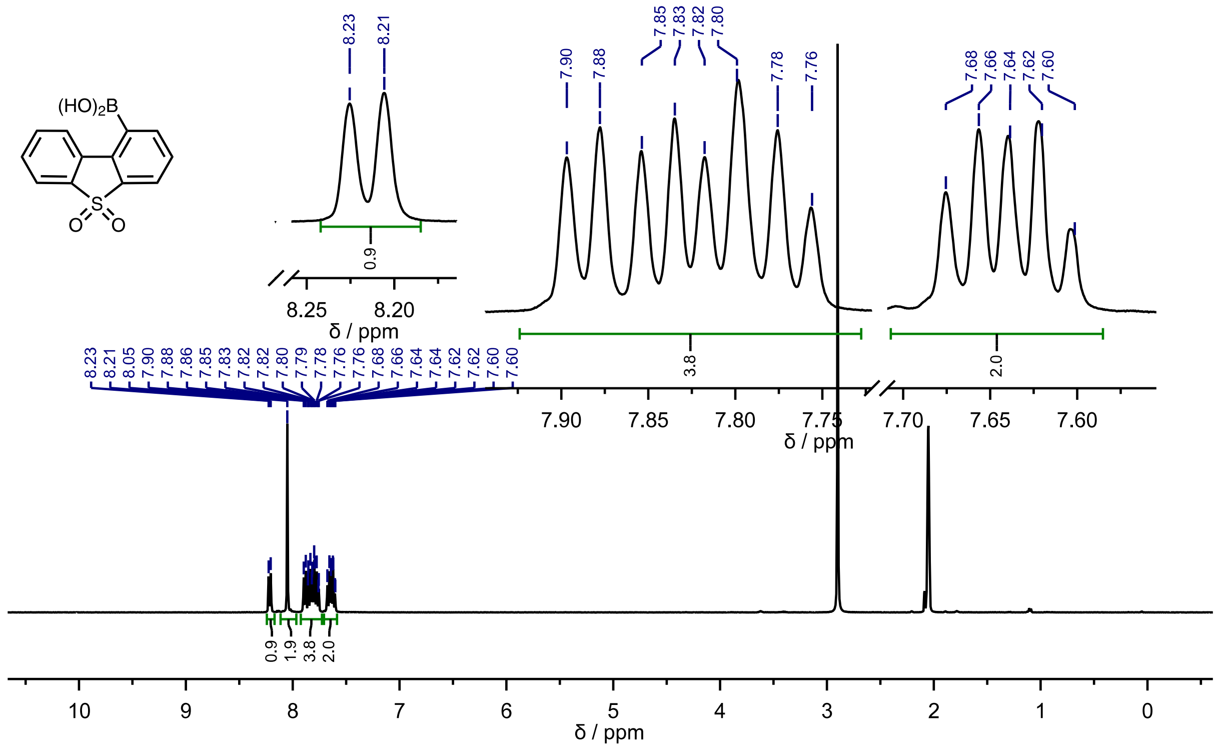

Figure S19. ^{1}H NMR spectrum (600 MHz, acetone-d_6) of compound **5**.

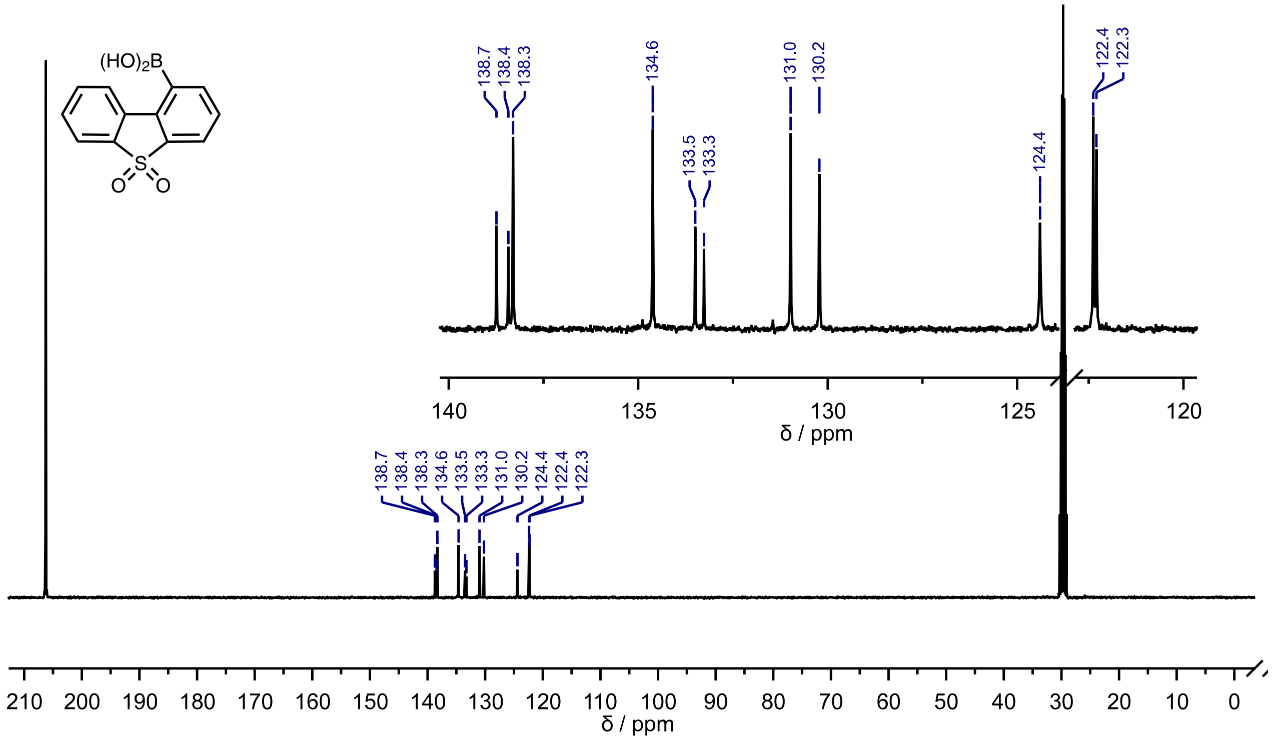

Figure S20. $^{13}C\{^{1}H\}$ NMR spectrum (151 MHz, acetone-d_6) of compound **5**.

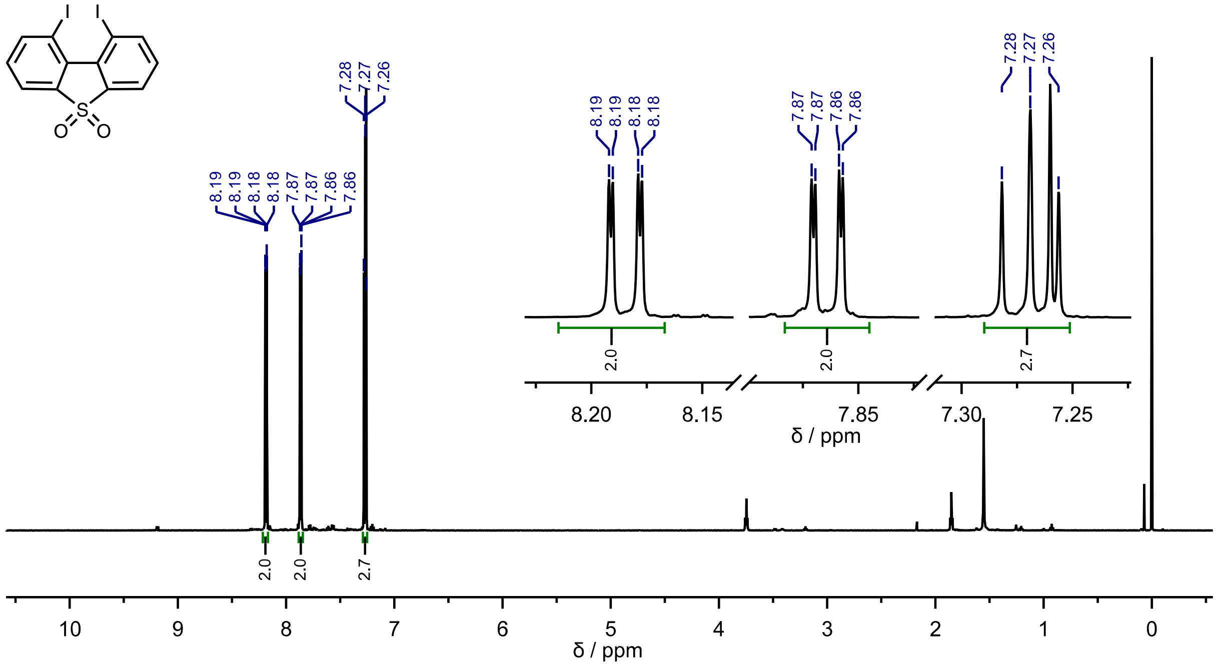

Figure S21. ^{1}H NMR spectrum (600 MHz, $CDCl_3$) of compound **6**.

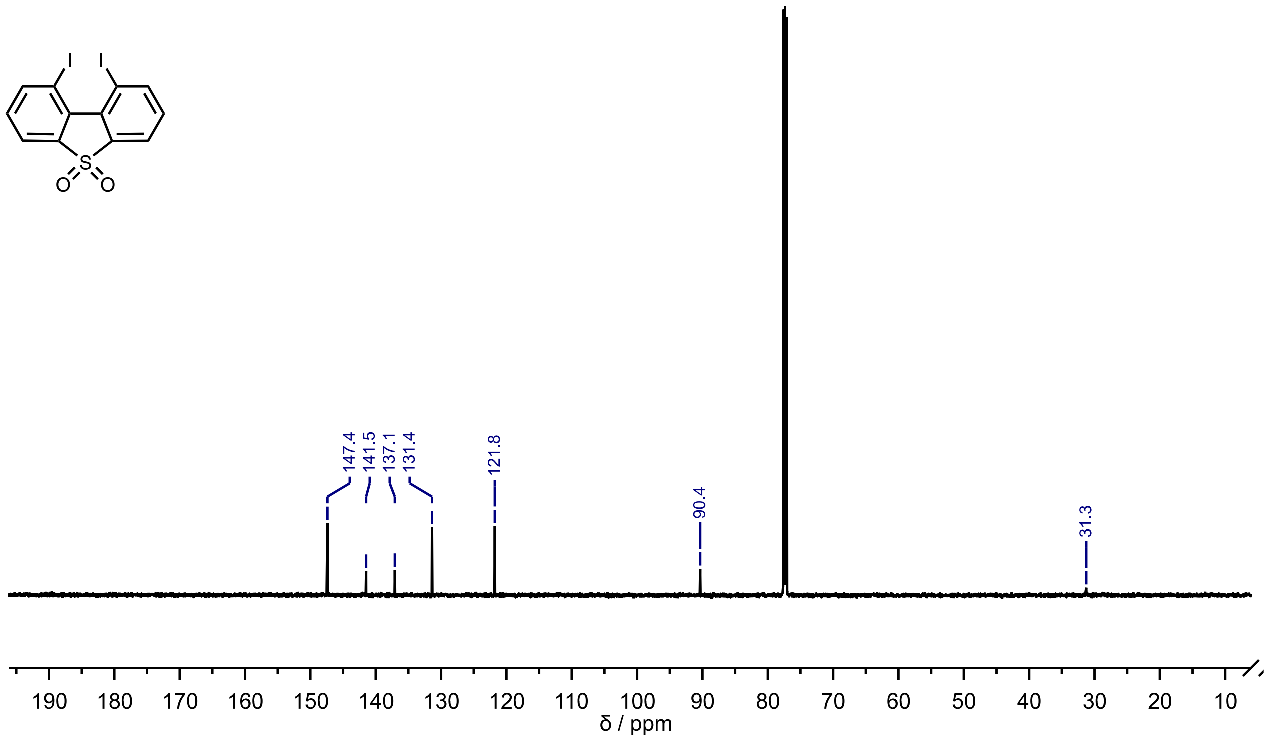

Figure S22. $^{13}C\{^{1}H\}$ NMR ($CDCl_3$, 151 MHz) spectrum of compound **6**.

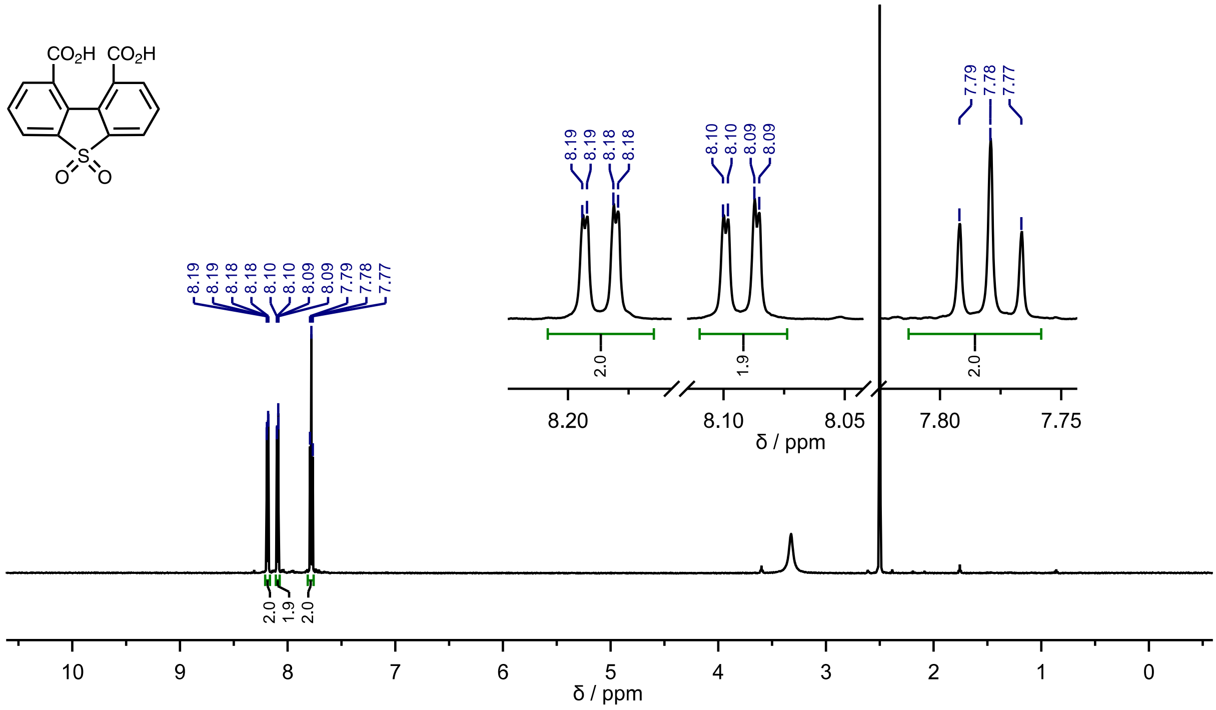

Figure S23. ^{1}H NMR spectrum (600 MHz, DMSO-d_6) of compound **7**.

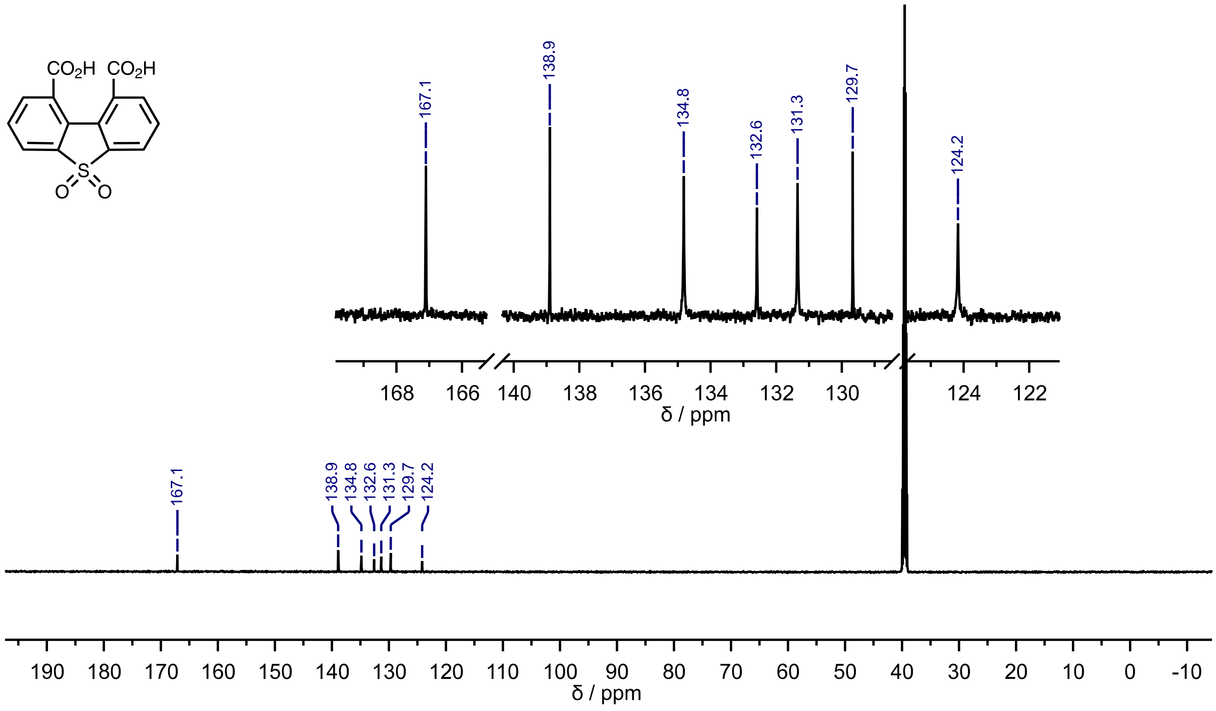

Figure S24. ^{13}C{^{1}H} NMR spectrum (151 MHz, DMSO-d_6) of compound **7**.

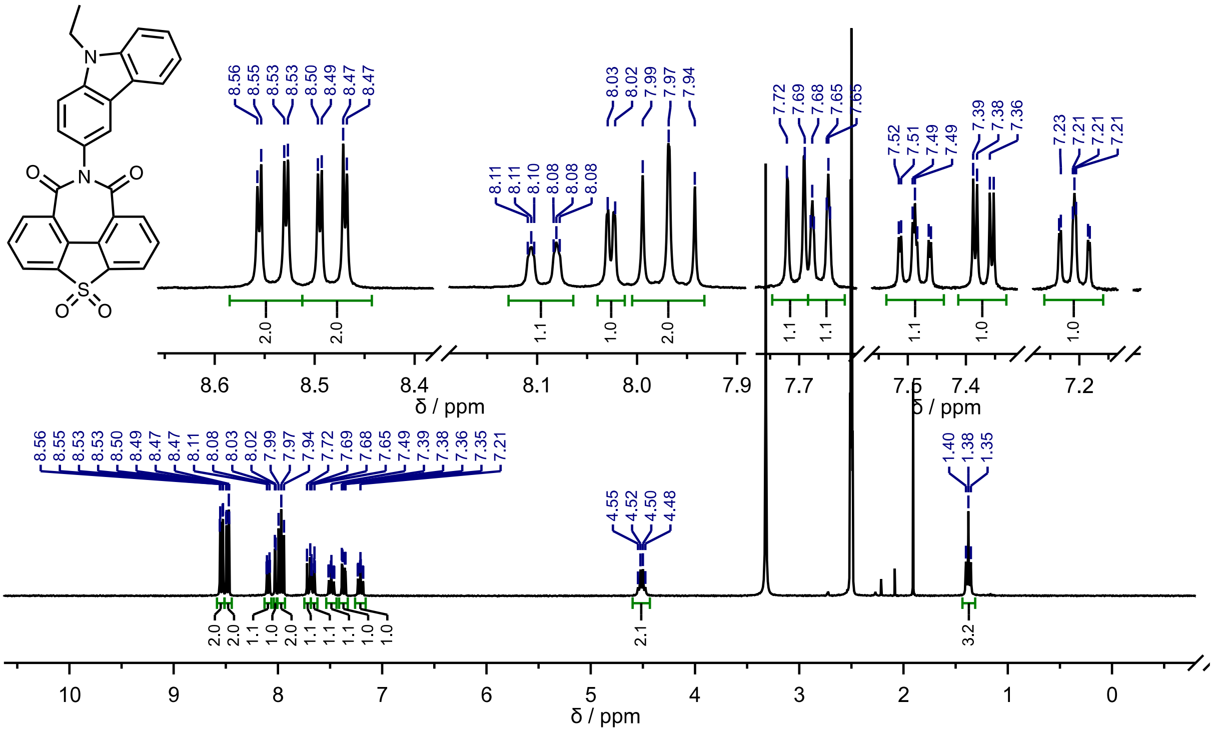

Figure S25. ^{1}H NMR spectrum (600 MHz, DMSO-d_6) of compound **8**.

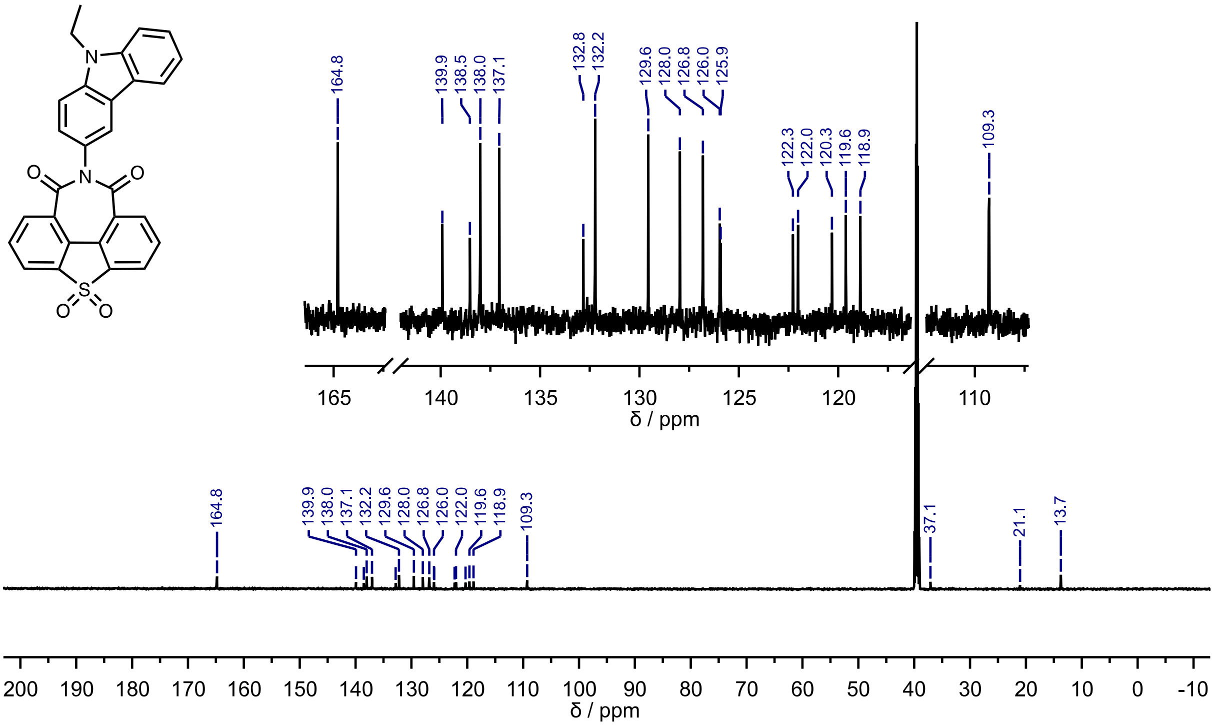

Figure S26. $^{13}C\{^{1}H\}$ NMR spectrum (151 MHz, DMSO-d_6) of compound **8**.

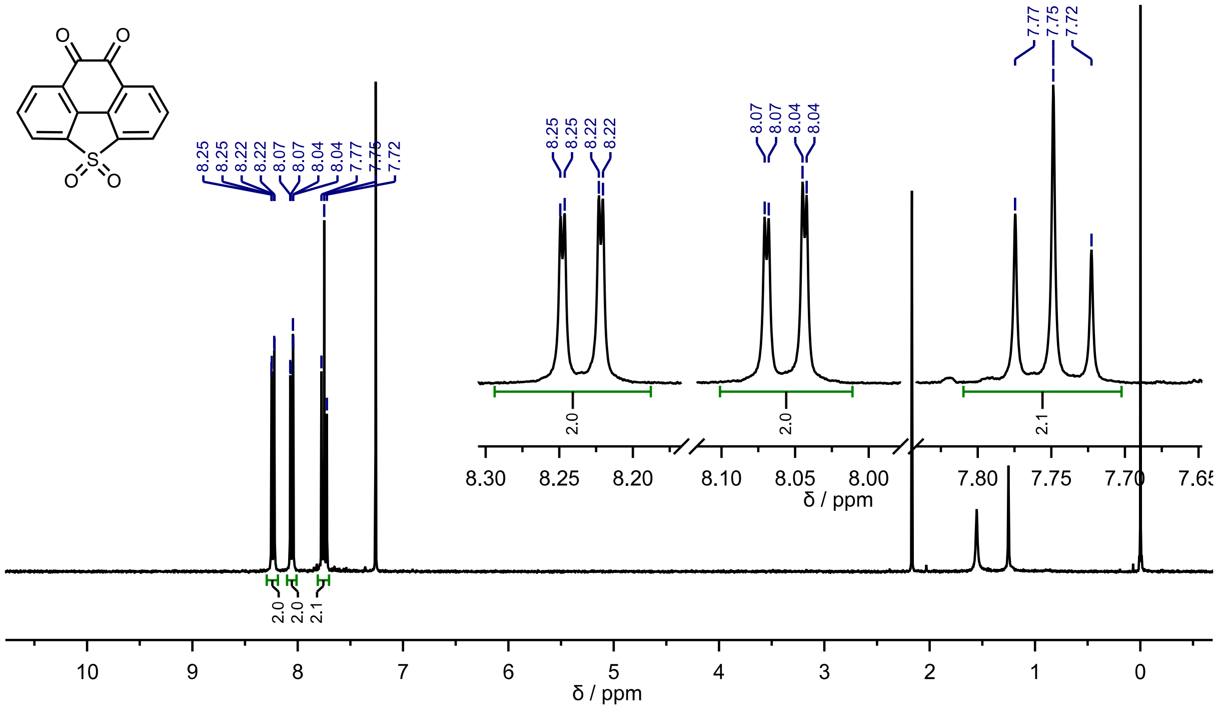

Figure S27. ^{1}H NMR spectrum (600 MHz, $CDCl_3$) of compound **9**.

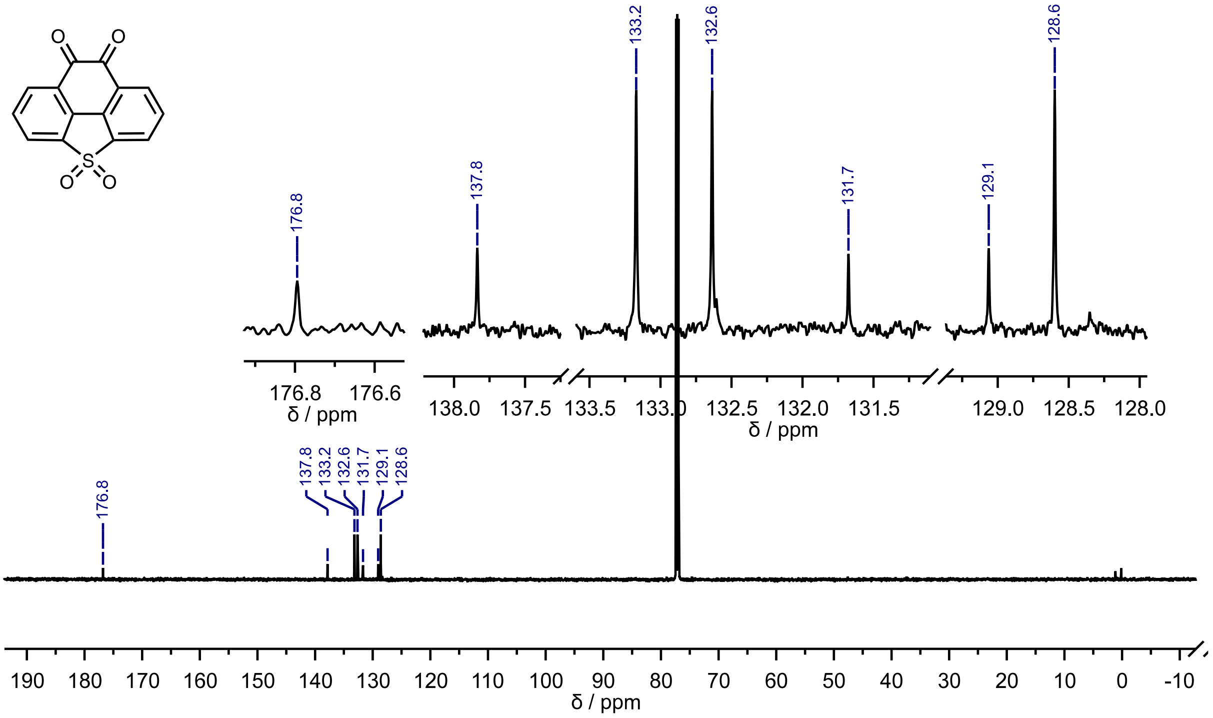

Figure S28. ^{13}C{^{1}H} NMR spectrum (151 MHz, $CDCl_3$) of compound **9**.

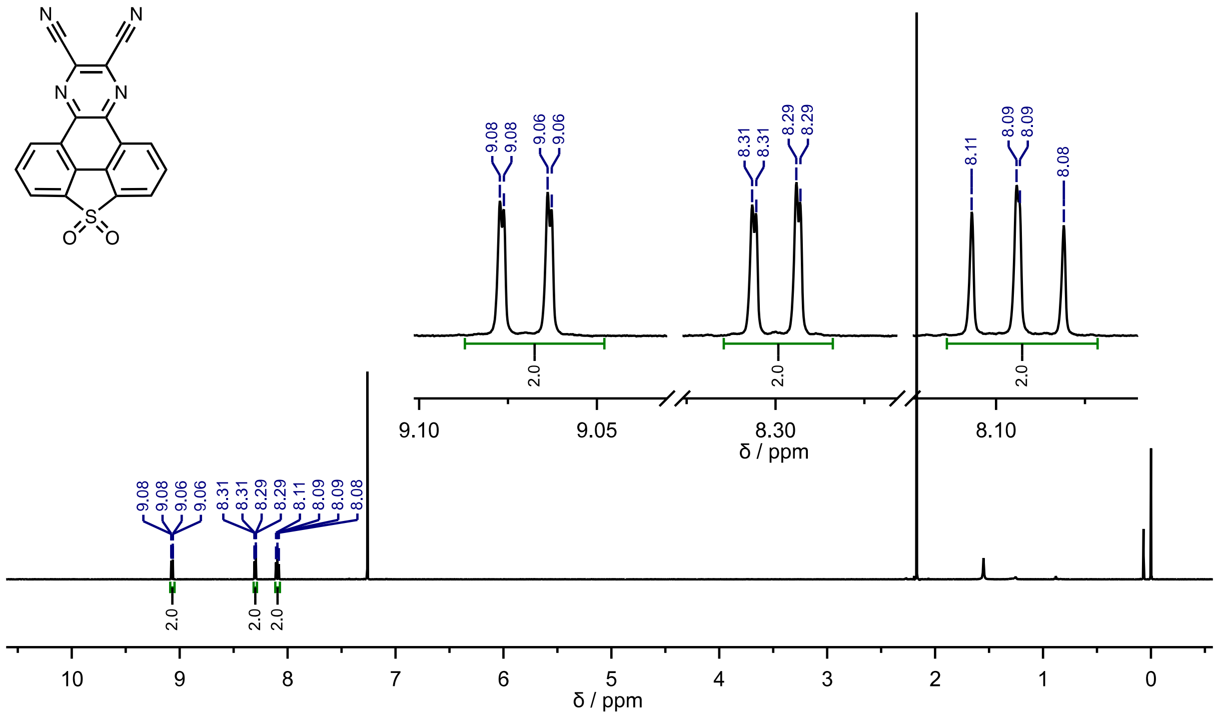

Figure S29. ^{1}H NMR spectrum (600 MHz, $CDCl_3$) of compound **10**.

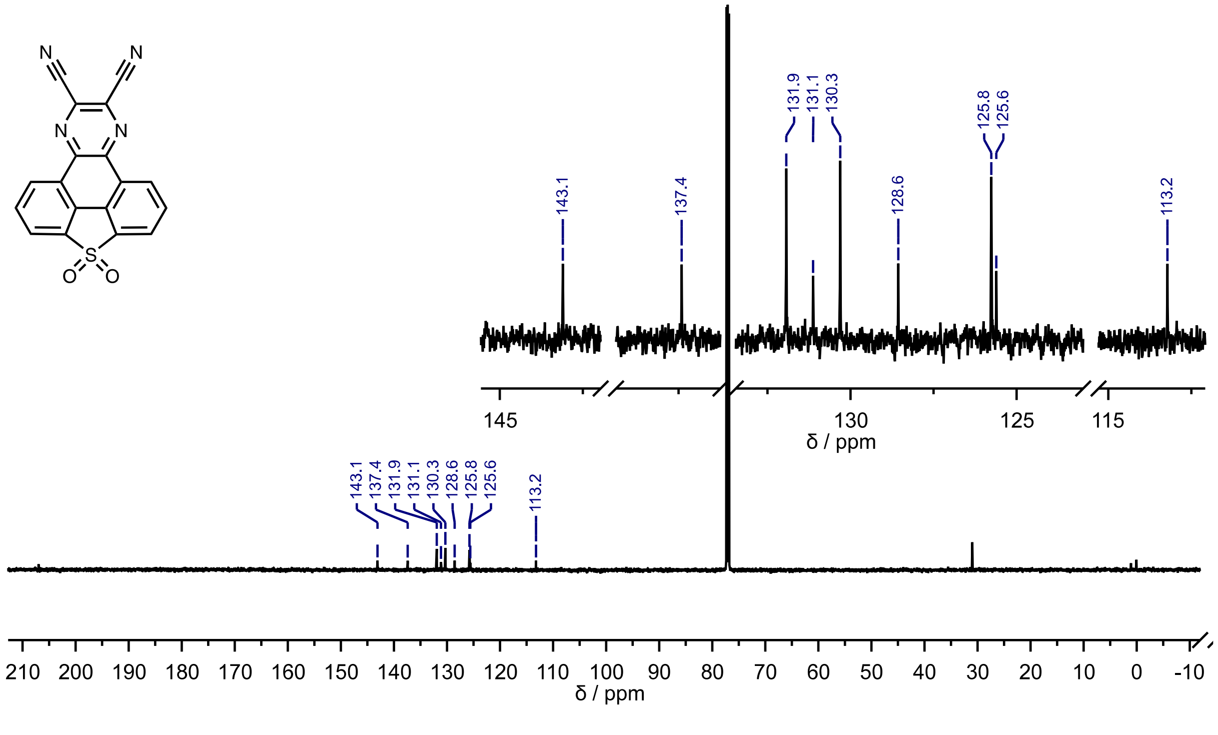

Figure S30. ^{13}C{^{1}H} NMR spectrum (151 MHz, $CDCl_3$) of compound **10**.

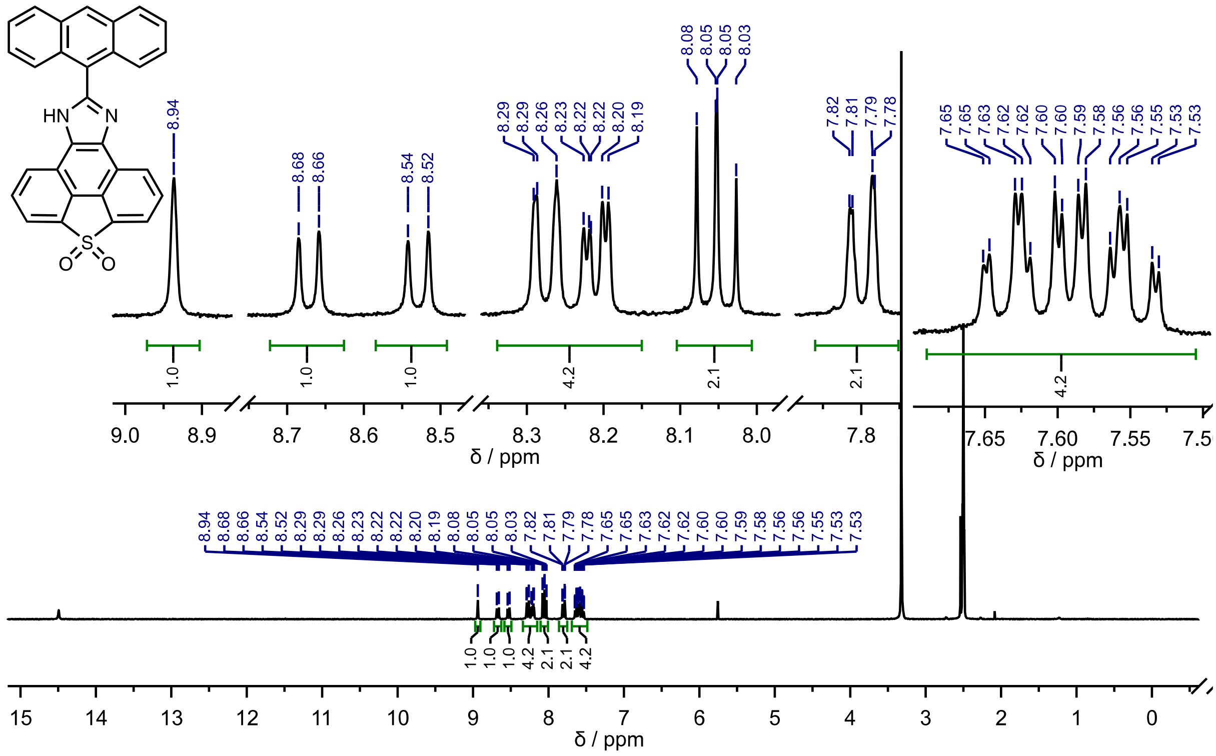

Figure S31. ^{1}H NMR spectrum (600 MHz, DMSO-d_6) of compound **11**.

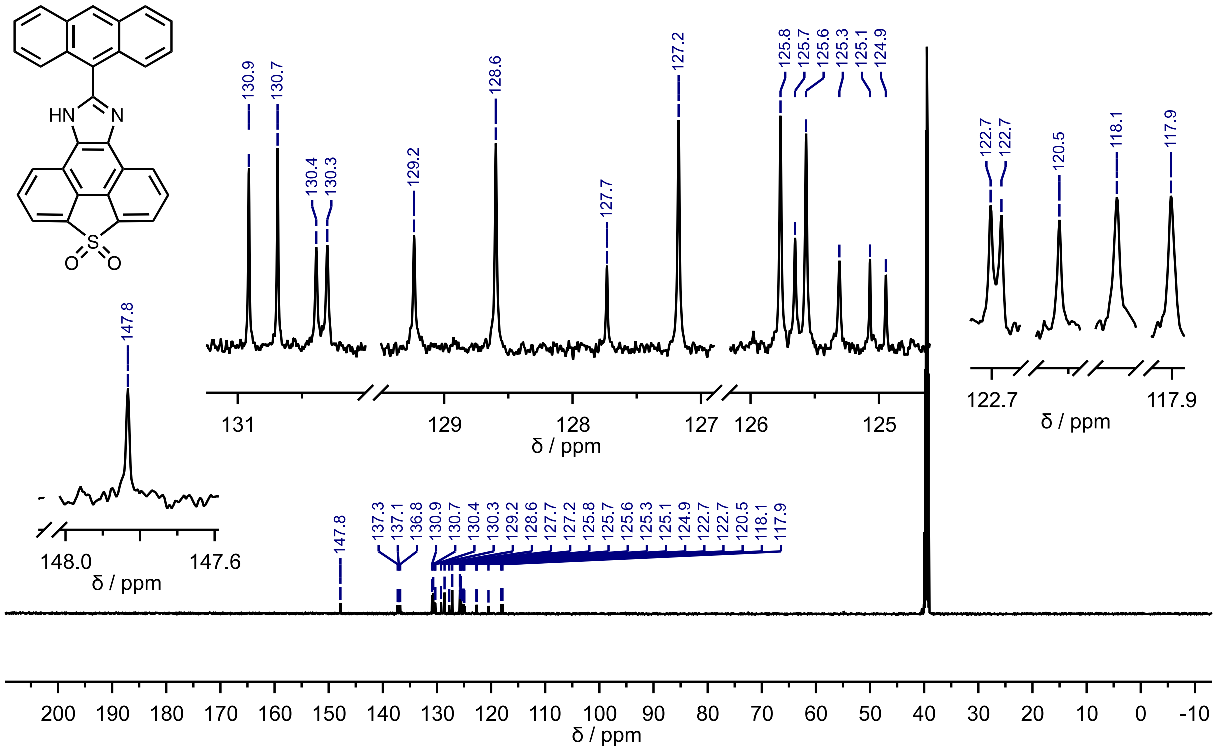

Figure S32. ^{13}C{^{1}H} NMR spectrum (151 MHz, DMSO-d_6) of compound **11**.

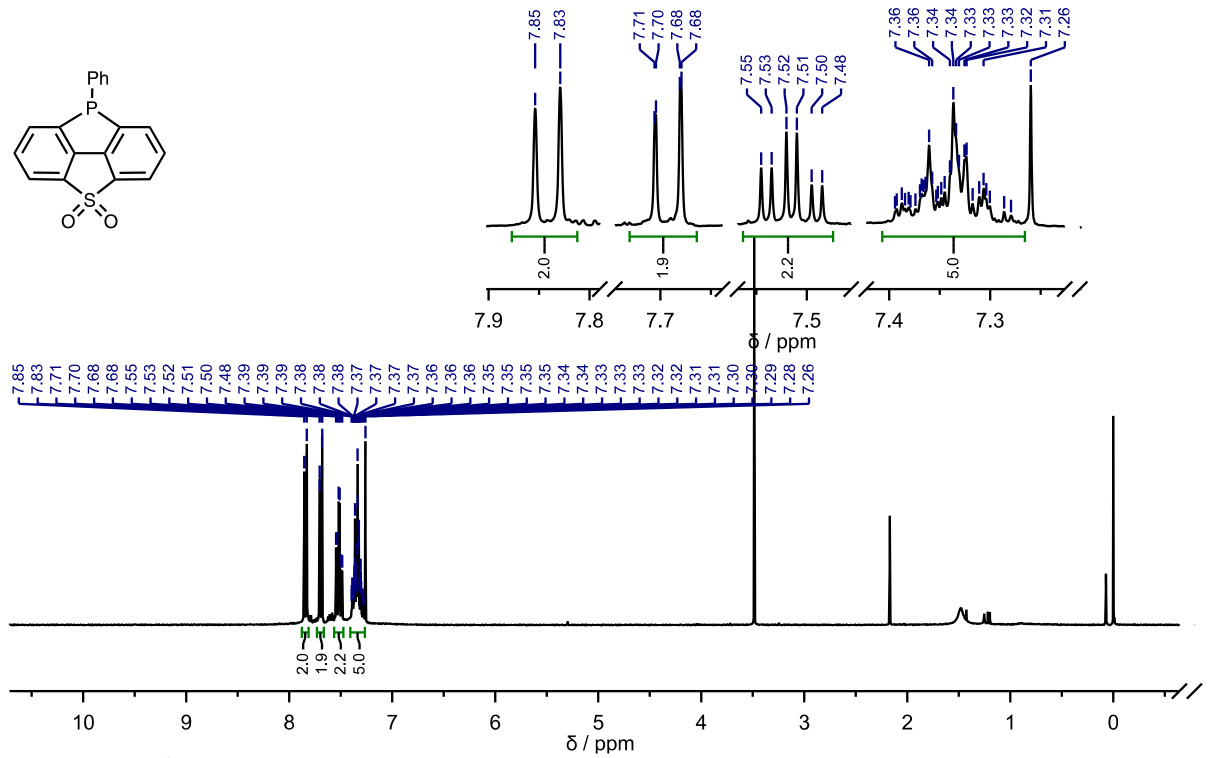

Figure S33. ^{1}H NMR spectrum (600 MHz, $CDCl_3$) of compound **12**.

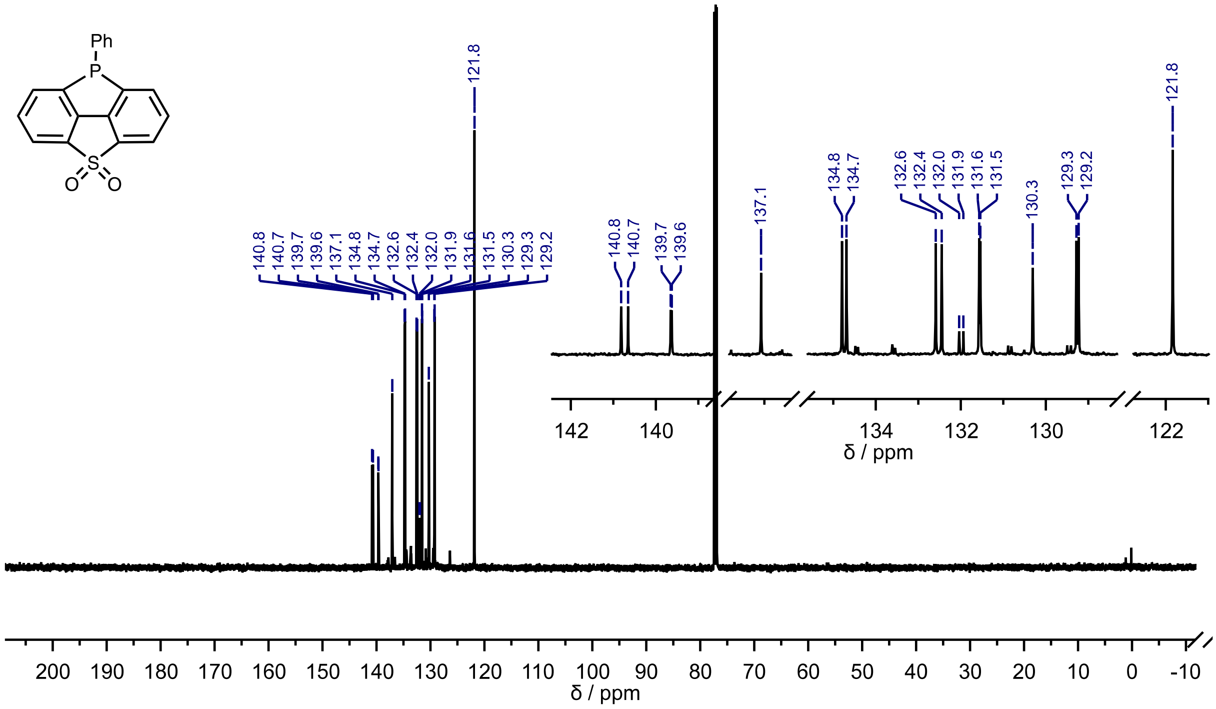

Figure S34. $^{13}C\{^{1}H\}$ NMR spectrum (151 MHz, $CDCl_3$) of compound **12**.

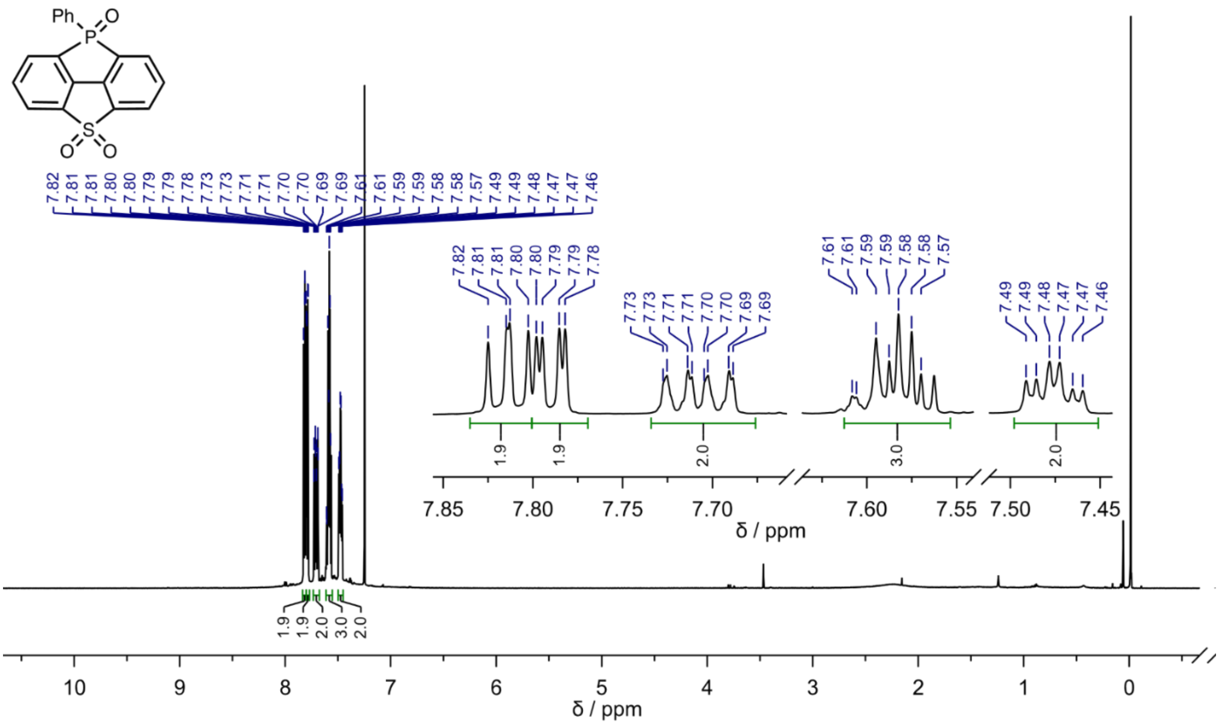

Figure S35. ^{1}H NMR spectrum (600 MHz, $CDCl_3$) of compound **13**.

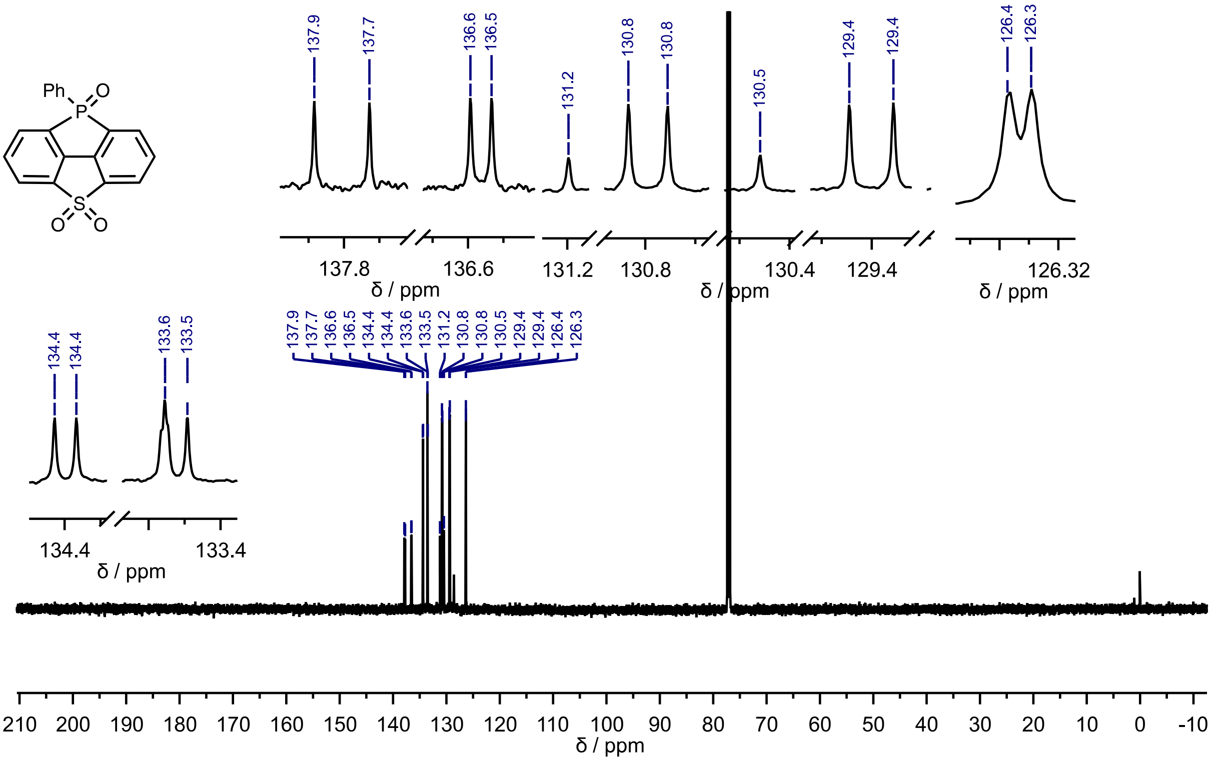

Figure S2. $^{13}C\{^{1}H\}$ NMR spectrum (151 MHz, $CDCl_3$) of compound **13**.

HRMS spectra

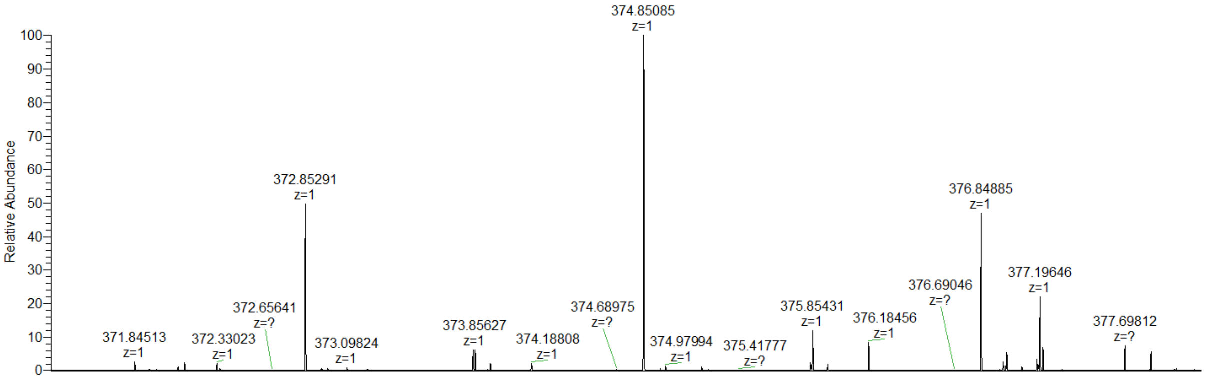

Figure S37. HRMS (ESI, positive ion mode) spectrum of compound **2**.

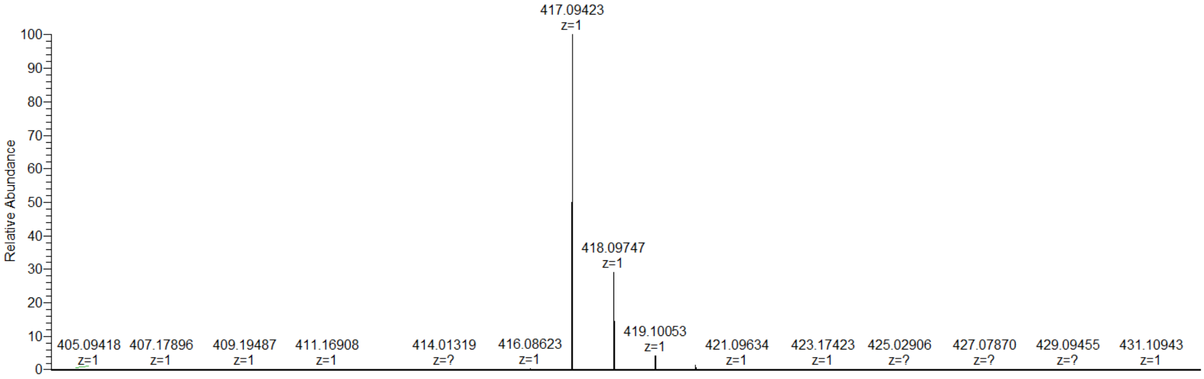

Figure S38. HRMS (ESI, positive ion mode) spectrum of compound **3**.

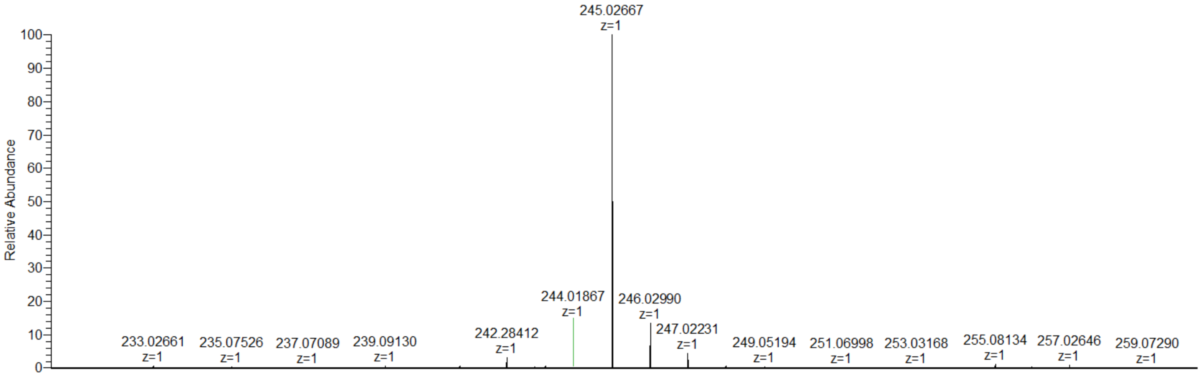

Figure S39. HRMS (ESI, positive ion mode) spectrum of compound **4**.

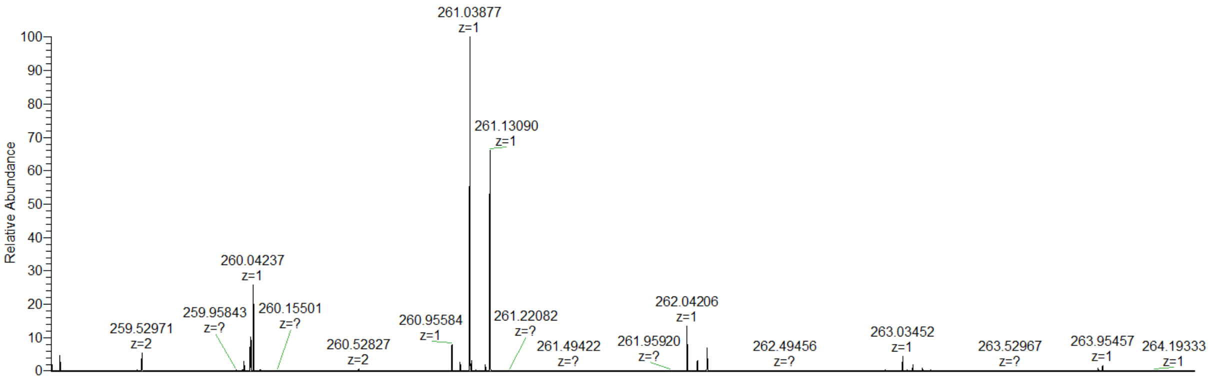

Figure S40. HRMS (ESI, positive ion mode) spectrum of compound **5**.

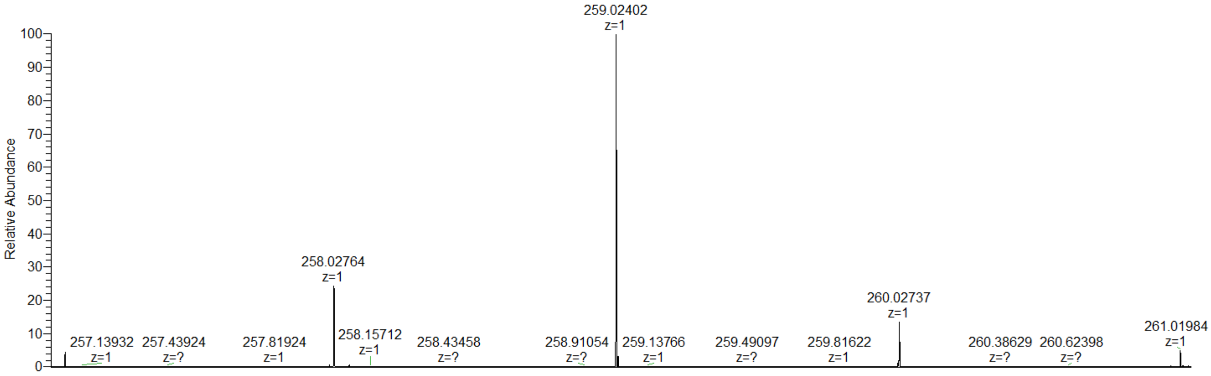

Figure S41. HRMS (ESI, negative ion mode) spectrum of compound **5**.

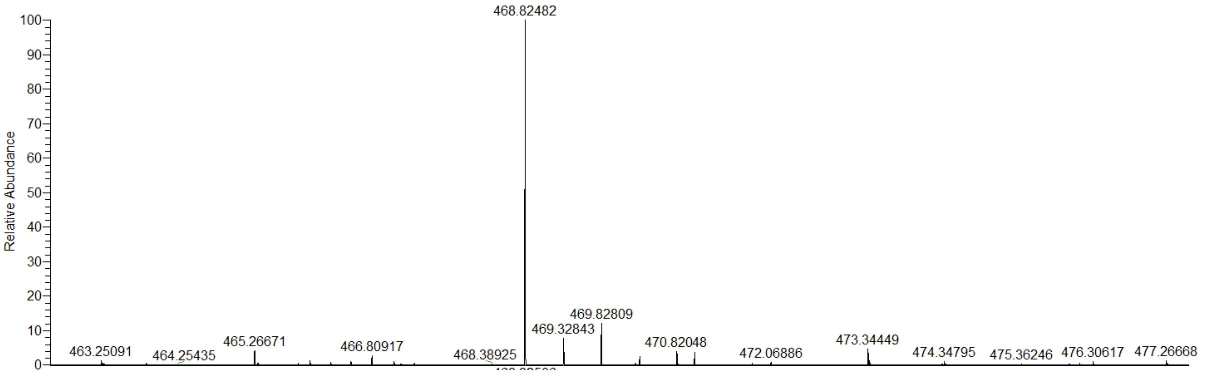

Figure S42. HRMS (ESI, positive ion mode) spectrum of compound **6**.

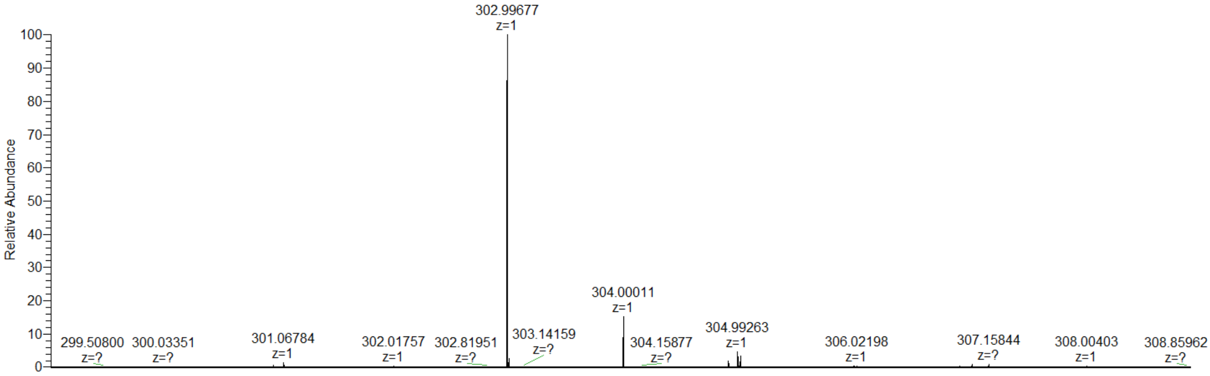

Figure S43. HRMS (ESI, positive ion mode) spectrum of compound **7** (negative ion mode).

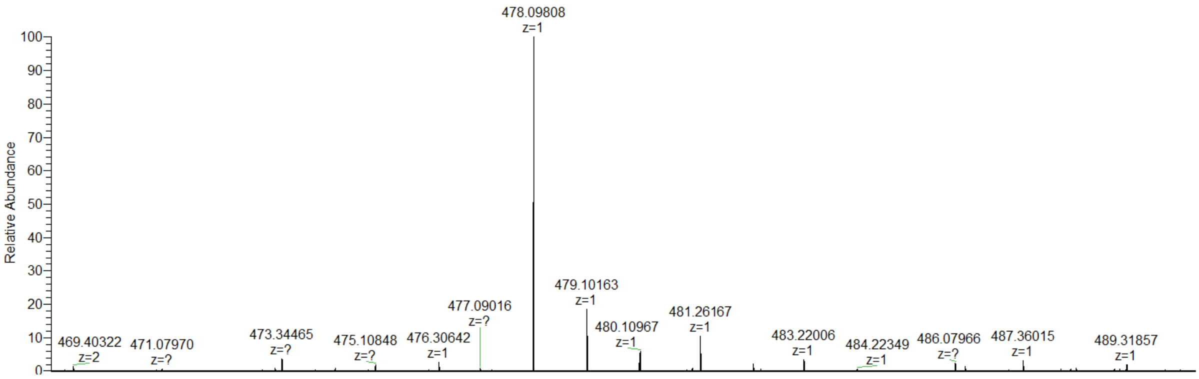

Figure S44. HRMS (ESI, positive ion mode) spectrum of compound **8**.

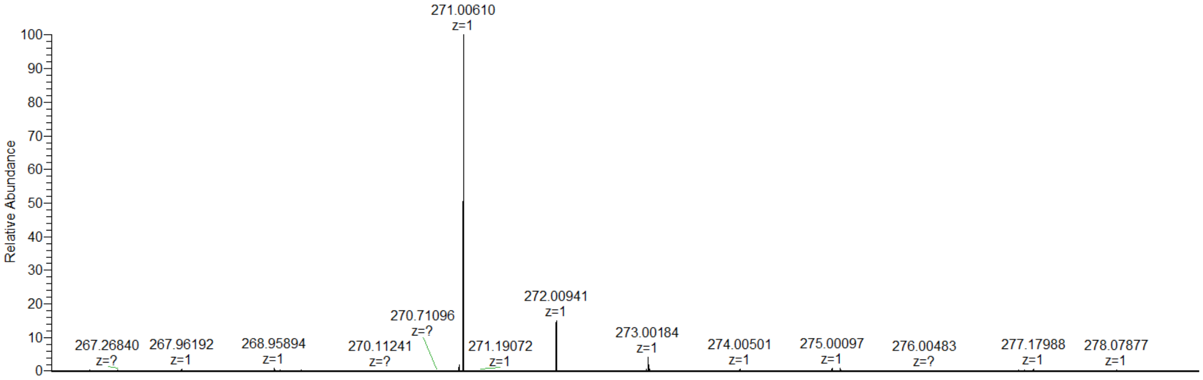

Figure S45. HRMS (ESI, positive ion mode) spectrum of compound **9**.

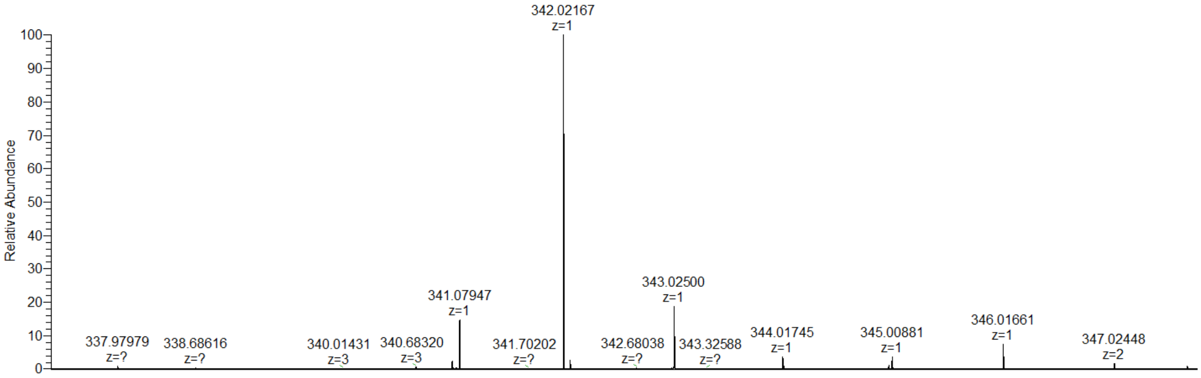

Figure S46. HRMS (ESI, negative ion mode) spectrum of compound **10**.

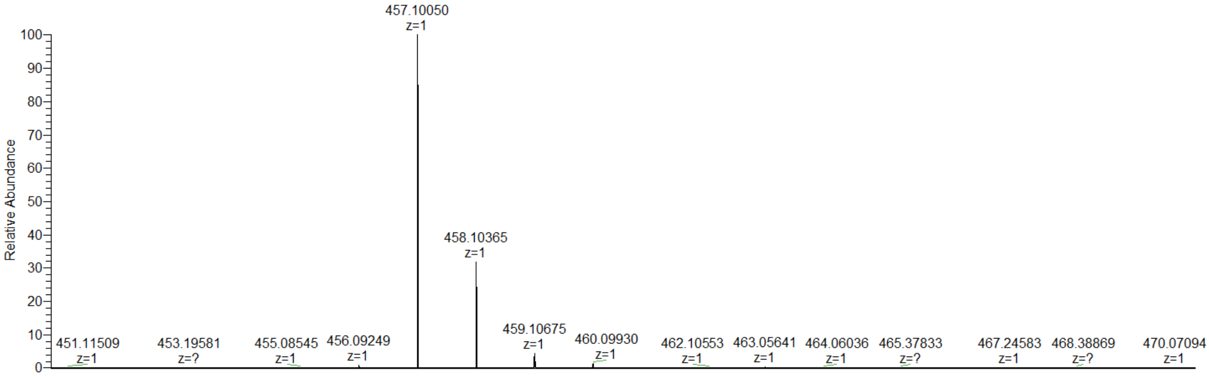

Figure S47. HRMS (ESI, positive ion mode) spectrum of compound **11**.

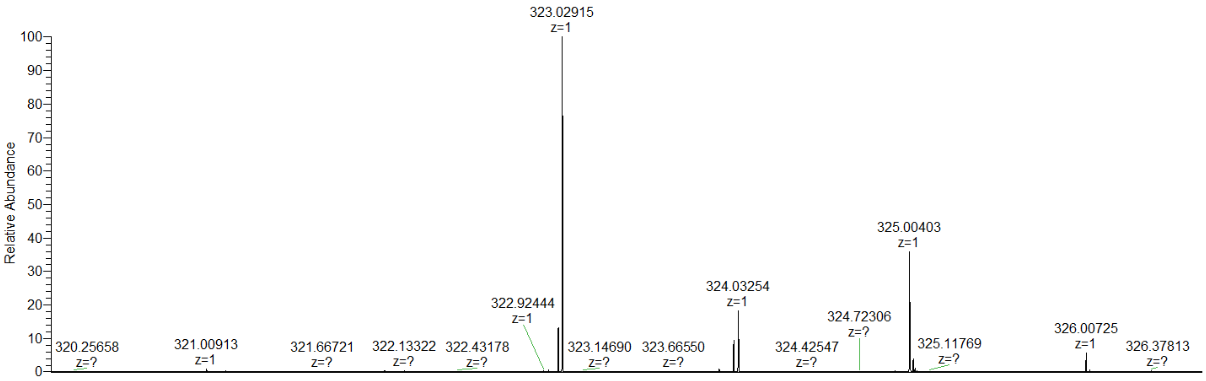

Figure S48. HRMS (ESI, positive ion mode) spectrum of compound **12**.

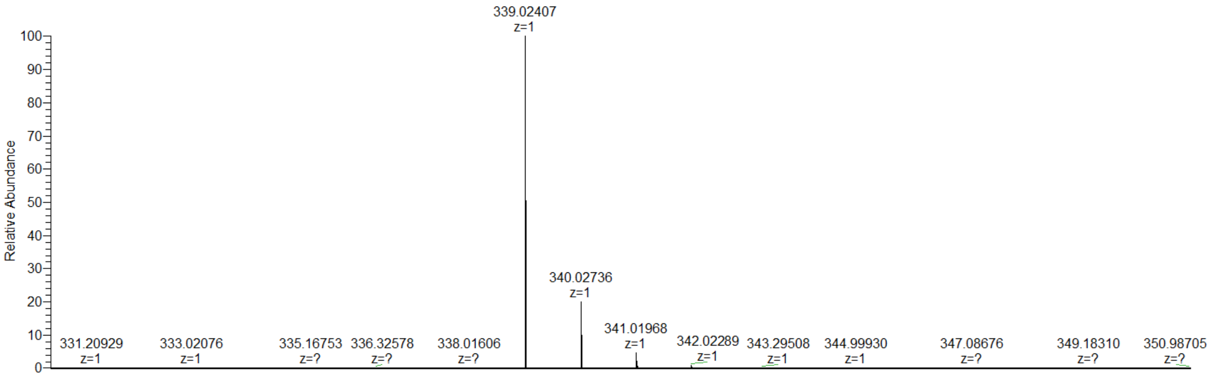

Figure S49. HRMS (ESI, positive ion mode) spectrum of compound **13**.
